# Sex and gender correlates of sexually polymorphic cognition

**DOI:** 10.1186/s13293-023-00579-8

**Published:** 2024-01-08

**Authors:** Louis Cartier, Mina Guérin, Fanny Saulnier, Ioana Cotocea, Amine Mohammedi, Fadila Moussaoui, Sarah Kheloui, Robert-Paul Juster

**Affiliations:** 1 Center on Sex*Gender, Allostasis, and Resilience, Research Center of the Montreal Mental Health University Institute, 7331, Rue Hochelaga, Montreal, QC H1N 3V2 Canada; 2 Department of Psychiatry and Addiction, University of Montreal, Montreal, QC Canada; 3 Department of Psychology, University of Montreal, Montreal, QC Canada; 4 School of Psychology, University of Ottawa, Ottawa, ON Canada

**Keywords:** Sexually polymorphic cognition, Sex differences, Sex hormones, LGBTQ+, Gender identity, Gender roles, Sexual orientation

## Abstract

**Background:**

Sexually polymorphic cognition (SPC) results from the interaction between biological (birth-assigned sex (BAS), sex hormones) and socio-cultural (gender identity, gender roles, sexual orientation) factors. The literature remains quite mixed regarding the magnitude of the effects of these variables. This project used a battery of classic cognitive tests designed to assess the influence of sex hormones on cognitive performance. At the same time, we aimed to assess the inter-related and respective effects that BAS, sex hormones, and gender-related factors have on SPC.

**Methods:**

We recruited 222 adults who completed eight cognitive tasks that assessed a variety of cognitive domains during a 150-min session. Subgroups were separated based on gender identity and sexual orientation and recruited as follows: cisgender heterosexual men (*n* = 46), cisgender non-heterosexual men (*n* = 36), cisgender heterosexual women (*n* = 36), cisgender non-heterosexual women (*n* = 38), gender diverse (*n* = 66). Saliva samples were collected before, during, and after the test to assess testosterone, estradiol, progesterone, cortisol, and dehydroepiandrosterone. Psychosocial variables were derived from self-report questionnaires.

**Results:**

Cognitive performance reflects sex and gender differences that are partially consistent with the literature. Interestingly, biological factors seem to better explain differences in male-typed cognitive tasks (i.e., spatial), while psychosocial factors seem to better explain differences in female-typed cognitive tasks (i.e., verbal).

**Conclusion:**

Our results establish a better comprehension of SPC over and above the effects of BAS as a binary variable. We highlight the importance of treating sex as a biological factor and gender as a socio-cultural factor together since they collectively influence SPC.

**Supplementary Information:**

The online version contains supplementary material available at 10.1186/s13293-023-00579-8.

## Plain Language Summary

Many studies show sex differences in cognitive abilities. In general, women outperform men in verbal tasks and fine motor skills, while men outperform women in spatial orientation and mental rotation tasks. These differences underlie research on sexually polymorphic cognition, a concept influenced by sex hormones (estradiol, progesterone, and testosterone) as well as birth-assigned sex. In addition to these biological factors, socio-cultural gender factors such as gender identity (the gender we feel and embody), gender roles (masculine and feminine expressions based on stereotypes), as well as sexual orientation are all known to influence cognition as well. We provide a broader understanding by accounting for both sex and gender factors. Our team recruited 222 adults separated into 5 sub-groups based on birth-assigned sex, gender identity, and sexual orientation. Each participant completed eight sexually polymorphic cognitive tasks. In this 150-min experimental protocol, saliva samples were collected before, during, and after the test to assess testosterone, estradiol, progesterone, cortisol, and dehydroepiandrosterone. Psychosocial variables were derived from self-report questionnaires. Results showed that spatial cognition was better explained by biological sex factors, while verbal cognition was better explained by socio-cultural gender factors. Taken together, our findings demonstrate the importance of considering sex-based and gender-based factors collectively and, respectively, when studying sex differences in cognition.

**Supplementary Information:**

The online version contains supplementary material available at 10.1186/s13293-023-00579-8.

## Highlights

- Sex differences in cognition have been extensively studied but remain unclear
- Incorporating sex and gender factors in cognitive studies is essential
- Inclusion of sexual and gender diversity further explain cognitive sex differences
- Contribution of each sex and gender factor depend on the cognitive function observed
- Spatial cognition appears more sexed, while verbal cognition appears more gendered.

**Supplementary Information:**

The online version contains supplementary material available at 10.1186/s13293-023-00579-8.

## Introduction

Sex differences in cognitive functioning have been extensively studied for decades [1–9]. To date, however, it is mainly birth-assigned sex that is the central point of comparison in this controversial field. Even if studies present sex differences, many men, women, and gender-diverse people perform in a wide variety of ways that go beyond sex as a binary variable. Many of these presumed sex differences are referred to as sexually polymorphic cognition (SPC).

SPC differ across the lifespan due in large part of hormonal variations influenced by reproductive factors and age-related declines in sex hormone concentrations [10–14]. SPC also presents itself across different countries, suggesting a strong biological driver that appears in part independent of cultural contexts [15–17]. And yet, some scholars argue that mixed results stem from focusing solely on biological aspects of sex [3, 18–20] Individual differences in SPC performance could be further understood by also assessing socio-cultural gender-based factors [21–23]. The current study assesses how birth-assigned sex, sex hormones, gender identity, gender roles, and sexual orientation uniquely influence SPC.

### Sex differences in cognition

Numerous studies show that females outperform males at perceptual speed tasks, verbal tasks, and fine motor skills [24–32]. Semantic verbal fluency (the ability to generate verbally words based on a specific semantic category), phonemic verbal fluency (the ability to generate verbally words based on a specific letter or phoneme), and verbal memory (the ability to memorize words and other abstractions involving language) are among those cognitive functions that have received the most attention [28, 33, 34]. Meta-analyses have shown effect sizes in verbal memory and verbal fluency tasks ranging between *d* = 0.27 and *d* = 0.34 [13, 29, 30]. The largest effect sizes are observed with the *California Verbal Learning Task* [35], with a mean of *d* = 0.48. Overall, studies tend to be mixed for verbal fluency, since several studies have not supported sex differences for this ability [36, 37] or reported different sex differences effects sizes depending on the nature of the verbal fluency task [30]. A recent meta-analysis attempted to investigate these mixed results and concluded that phonemic fluency (*d* = 0.13) generally yields stronger female advantages than semantic fluency (*d* = 0.02) [30].

In general, males outperform females in mental rotation, navigation tasks, and spatial orientation [38–44]. In contrast to verbal fluency, mental rotation (the ability to rotate a three-dimensional object mentally) has consistently shown better performances among males when compared to females with means ranging between 0.57 and 0.90 [5, 45, 46]. Moreover, performance on *Judgement Line Orientation task* [47], a visuospatial judgement task that does not require motor skills, presents similar consistency ranging between 0.65 and 0.85 [48, 49]. Smaller, but still medium effect sizes have been observed with spatial memory tasks, varying across 0.58 and 0.73 [50, 51].

Beyond birth-assigned sex, there are strong indicators that sex hormones are associated with SPC. Among males and females, circulating testosterone can predict performance on visuospatial tasks [52–56]. Indeed, better visuospatial performance is often reported when endogenous testosterone levels are higher [1, 57, 58]. This is further influenced by menstrual cycling of estradiol and progesterone concentrations. Scientific literature seems to discern a portrait of the effect of sex hormones on cognitive performances following a linear relationship. Indeed, females are better at female-dominant cognitive tasks such as manual coordination, verbal fluency, and verbal memory during the mid-luteal phase, characterized by rises of estrogen and progesterone [59–62]. By contrast, post-menopausal females experience a rise in testosterone and a decrease in estrogens that collectively corresponds to decreased performance in verbal memory performance but preserve their abilities when undergoing estrogen replacement therapy [63–66]. Similarly, older males receiving exogenous testosterone therapy have better performance on spatial memory tasks [67, 68], while those with higher endogenous estradiol concentrations show better verbal memory [69, 70].

Although scientific literature shows evidence of linear relationship between androgen levels in spatial tasks and estrogen levels in verbal tasks, other studies suggest that SPC performances could be expressed in relation to sex hormones following an inverted U-shaped curve [71–73]. Indeed, better performance on the mental rotation task was recently observed in men with lower testosterone levels and women with higher levels [74–77]. Furthermore, the relationship between memory task performance and estradiol levels follows a similar pattern [78]. That said, better cognitive performance might occur when estradiol and testosterone levels are balanced [79]. The relationship between sex hormones and cognition might not be linear and still requires further investigation. Cognitive performance also differs across menstrual cycle depending on contraceptives [80]. Naturally cycling females show stronger performance on verbal fluency task than oral contraceptives users, who present lower estradiol and progesterone levels [81, 82]. Lower mental rotation performance among naturally cycling females are observed when compared to males and oral contraceptive users [60, 83–85].

Sex hormones interfere with stress hormones (e.g., cortisol) and can also affect SPC in ways that differ markedly among men, women, and gender-diverse people. Importantly, increased secretion of stress hormones has a negative effect on cognitive functions and should therefore be adjusted for in SPC research [86]. Moreover, the androgen dehydroepiandrosterone (DHEA) that acts as an antagonist of cortisol has been considered only in a few cognitive studies [87] primarily examining its therapeutic effects on cognitive decline in post-menopausal women, but showing no evidence of benefits [88–90]. The link between DHEAS (its sulfate form) and cognitive decline have also been investigated in some studies, where both low and high level of DHEAS were associated with poorer cognitive functioning among men, but not among women [91, 92]. To date, transgender and gender-diverse people have received little attention in the study of the link between hormones and cognition but may provide further insights into neuroendocrine mechanisms of SPC [93].

### Gender-based factors in cognition

Beyond birth-assigned sex (male/female) and sex hormones, socio-cultural gender refers to attitudes, affiliations, identities, and behaviors that also impact SPC [7]. For example, androgynous people who endorse gender role profiles of high masculinity and high femininity seem to perform better at verbal tasks than women who otherwise outperform other groups [94, 95]. Moreover, higher prenatal androgen exposure may cause females to seek out male-typical gendered behaviors (e.g., playing with construction sets, playing video games, or practicing sports) that are likely to enhance spatial cognition [96].

*Gender identity* refers to a person’s innermost concept of themselves as male, female, non-binary, agender, and a diversity of identities that can be the same or different from one’s physical sex [97]. Indeed, gender identity is not always binary (girl/woman, boy/man) or fixed [98]. Gender identity is expressed along a continuum and can be dynamic across time [99]. Cognitive performance of people who identify as transgender appears to be more consistent with the gender to which they self-affirm, rather than with their birth-assigned sex [93, 100–104]. Cisgender men (people who identify as men and are assigned male sex at birth) and transmasculine people present similar mental rotation skills, both performing better than cisgender women [105–107]. In parallel, higher masculine and lower feminine self-concepts are associated to better performance on spatial cognitive task [94, 95, 108]. More research is needed to better understand how gender identity shapes cognition among gender non-binary communities [93, 109] that may not identify with masculine or feminine binaries.

Cisgender and transgender people alike internalize socio-cultural definitions of *gender roles* that surround them across lifespan development. Gender roles are defined as the different expectations that individuals, groups, and societies at a larger scale have towards individuals, based on their sex and based on the respective society’s values and beliefs about gender [110]. There is a diversity of ways people understand, experience, and express their gender, whether influenced by those gender roles they assimilate and the institutional gender expectations that surround them [111]. Gender roles at the individual-level become gender norms that are perpetrated by gendered “standards” regarding how people behave. This encourages and enforces people to behave in ways that conform to idealized conceptions of gender expression [112, 113]. Some studies have mentioned that performance on cognitive tasks tend to be better if one’s gender self-concept is coherent with gendered social expectations of a specific gendered task [114, 115].

Orthogonal to sex and gender is sexual orientation that is reported as one of the most important factors influencing SPC [116, 117]. Sexual orientation refers to an enduring pattern of emotional, romantic, and/or sexual attractions to men, women, both, no gender, or all genders [98]. In the SPC literature, sexually diverse people show gender inversions in cognitive performance [118–120]. For example, gay men perform better at verbal fluency than heterosexual men, a pattern similar to the better performance seen among heterosexual women [121, 122]. Moreover, gay men perform worse at spatial navigation and mental rotation tasks than heterosexual men [121, 123, 124].

A similar pattern of « cross-sex shifting» seems to be present with SPC performance among sexually diverse women. Indeed, lesbians’ performances in spatial perception and in mental rotation are better than heterosexual women [124]. This result is a part of a meta-analysis reporting that lesbian women’s cognitive performance matches heterosexual men, but only on male-typed tasks (mental rotation, spatial navigation, and spatial perception) [125]. From these results, this idea of cross-sex shift has been put forward by many researchers, but needs future investigations [119, 126]. Taken together, individual differences in SPC are therefore influenced by a multitude of sex- and gender-based factors in addition to sexual orientation that need to be further studied collectively. In accordance, our objective here is to delineate the contributions of these factors collectively and additively as predictors of SPC.

### Objectives and hypothesis

The literature on SPC is vast but often mixed and studied in disciplinary silos. We suspect that methodological differences regarding hormone measurement and adjustments have led to numerous discrepancies [12, 127, 128]. Moreover, many studies ignore socio-cultural gender-based factors as sources of hormonal variation. Most importantly, many studies regarding SPC have focused on single sex-based factor or studied single gender-based factor at a time. Since studies have shown the collective importance of sex-based factors (i.e., birth-assigned sex and sex hormones) and gender-based factors (i.e., gender identity, gender roles, and sexual orientation), a transdisciplinary approach could provide a better understanding of SPC among men, women, and gender-diverse people.

The first objective of this study is to assess if the typical sex/gender differences in a comprehensive battery of classic tasks assessing cognitive functioning can be replicated such that the biological and socio-cultural contribution can be further examined. Over and above sex as a binary variable, the second objective is to determine the effect of multiple variables such as sex hormones, gender identity, gender roles, and sexual orientation on SPC and what best explain these between-sex differences or within gender diversity.

Our main analyses use hierarchical regressions to model SPC using biological sex-based factors followed by socio-cultural gender-based factors and sexual orientation thereafter. First, we hypothesize that cognitive performance will be sexually polymorphic with birth-assigned females outperforming birth-assigned males in verbal and fine motor skills tasks, while birth-assigned males will outperform females in mental rotation, visuospatial perception, and spatial memory. Second, we expect sex hormones (testosterone, progesterone, estradiol) to further explain variance in SPC over and above birth-assigned sex. Third, we hypothesize that congruence of one’s gender identity, gender roles, and sexual orientation with a given SPC task will further explain individual differences over and above birth-assigned or sex-based factors (e.g., sex hormones). And fourthly, we hypothesize that sex hormones (estradiol, progesterone, and testosterone) and gender identity will present themselves overall as the strongest predictors of cognitive functioning, over and above gender roles, birth-assigned sex, and sexual orientation.

## Methods

First of all, it is worth noting that when sex assigned at birth was referred, the terms "male" and "female" are used. When referring to gender identity, the terms "man", "women" and "gender diverse" are used. However, for the sake of clarity, the terms "cisgender man" and "cisgender woman" have been used interchangeably with the terms man and woman.

### Design and participants

This cross-sectional and quasi-experimental paradigm recruited *N* = 222 cisgender men, cisgender women, and gender-diverse people (e.g., non-binary, gender fluid, genderqueer, transgender people) between the ages of 18 and 69 (*M* = 27.92; SE ± 8.97). Since sex hormones are a major component of this project, participants from a wide age range were recruited from 18 years of age and older. Given hormonal variations across the lifespan, this age diversity was chosen to help us investigate endocrine effects on cognitive abilities. Except age, this study presented no other exclusion criteria. We chose liberal criteria to maximize representation of people from sexual and gender diversity, who often experience more stigma and stress that can exacerbate health conditions [129]. In accordance, factors that could have been used as exclusion criteria were treated as potential confounders. Participants were living in the greater Montreal area and needed to be fluent in either French or English.

Three virtual recruitment posters were developed to recruit from three populations: cisgender and heterosexual individuals, sexually diverse individuals (who were non-heterosexual), and gender-diverse people. Recruitment was primarily done via Facebook posts on lesbian, gay, bisexual, transgender, and queer (LGBTQ+) community groups, university community groups, and partnership with LGBTQ+ organizations.

Sample descriptive statistics are summarized in Table 1 as a function of gender identity according to demographics, socio-cultural gender variables, lifestyle behaviors, contraception and menstruation, and general health. Prior to conducting our study, we engaged in a participatory practice [130] with gender-diverse communities. Our team conducted semi-structured qualitative interviews with 33 gender-diverse people prior to testing. We identified health and wellness needs of this community and verified whether our research methodology and variables considered spoke to the concerns of this community that have been underrepresented.

**Table 1:** Descriptive statistics and groups differences

Characteristics	Sample	Cismen	Ciswomen	Gender diverse	*p*
***N***	222	82	74	66	–
*Demographic*					
Age, M (SE)^a^	27.92 (8.98)	29.88 (10.88)^c^	26.49 (8.49)^b^	27.11 (6.13)	0.041
Race/ethnicity					0.053
White, *n* (%)	175 (78.8)	56 (68.3)^e^	66 (89.2)^f^	53 (80.3)^e,f^	–
Black, *n* (%)	8 (3.6)	6 (7.3)^e^	2 (2.7)^e^	0 (0)^e^	–
Asian, *n* (%)	6 (2.7)	2 (2.4)^e^	1 (1.4)^e^	3 (4.5)^e^	-
Mixed, *n* (%)	15 (6.8)	9 (11.0)^e^	3 (4.1)^e^	3 (4.5)^e^	–
Maghrebian, *n* (%)	12 (5.4)	7 (8.5)^e^	2 (2.7)^e^	3 (4.5)^e^	–
Hispanic, *n* (%)	5 (2.3)	2 (2.4)^e^	0 (0)^e^	3 (4.5)^e^	–
Indigenous, *n* (%)	1 (0.5)	0 (0)^e^	0 (0)^e^	1 (1.5)^e^	–
Mother tongue					0.022
French, *n* (%)	169 (76.1)	66 (80.5)^e,f^	61 (82.4)^f^	42 (63.6)^e^	–
English, *n* (%)	22 (9.9)	5 (6.1)^e^	3 (4.1)^e^	14 (21.2)^f^	–
Bilingual, including French, *n* (%)	10 (4.5)	3 (3.7)^e^	3 (4.1)^e^	4 (6.1)^e^	–
Others, *n* (%)	21 (9.5)	8 (9.8)^e^	7 (9.5)^e^	6 (9.1)^e^	–
Occupational status					0.034
Workers, *n* (%)	106 (47.7)	45 (54.9)^e^	34 (45.9)^e^	27 (40.9)^e^	–
Students, *n* (%)	92 (41.4)	28 (34.1)^e^	37 (50.0)^e^	27 (40.9)^e^	–
Neither workers nor students, *n* (%)	24 (10.8)	9 (11.0)^e,f^	3 (4.1)^f^	12 (18.2)^e^	–
Working hours/week, *M* (SE) (only for workers)	30.57 (15.55)	30.98 (16.39)	31.79 (16.49)	28.33 (13.01)	0.674
Studying hours/week, *M* (SE) (only for students)	24.38 (13.66)	21.75 (14.73)	24.14 (13.47)	27.44 (12.64)	0.303
Ratio of men/women at work/school	1.24 (1.52)	1.64 (1.70)^c^	0.93 (1.00)^b^	1.12 (1.69)	0.013
Gender diversity at work/school, % (SE)	7.43 (7.83)	5.66 (6.96)^d^	6.81 (7.21)^d^	10.34 (8.79)^c,d^	0.001
Socioeconomics					
Education, years in school, *M* (SE)	16.44 (2.71)	16.61 (3.00)	15.89 (2.40)	16.83 (2.59)	0.092
Civil status					0.293
Single, *n* (%)	117 (52.7)	35 (42.7)^e^	44 (59.5)^e^	38 (57.6)^e^	–
In a relationship, *n* (%)	82 (36.9)	37 (45.1)^e^	26 (35.1)^e^	19 (28.8)^e^	–
Married, *n* (%)	11 (5.0)	5 (6.1)^e^	2 (2.7)^e^	4 (6.1)^e^	–
Divorced, *n* (%)	10 (4.5)	5 (6.1)^e^	2 (2.7)^e^	3 (4.5)^e^	–
Missing, *n* (%)	2 (0.9)	0 (0.0)	0 (0.0)	2 (3.0)	–
Relationship preference					< 0.001
Monoamorous, *n* (%)	133 (59.9)	60 (73.2)^e^	50 (67.6)^e^	23 (34.8)^f^	–
Polyamorous, *n* (%)	48 (21.6)	16 (19.5)^e^	5 (6.8)^e^	27 (40.9)^f^	–
Missing, *n* (%)	41 (18.5)	6 (7.3)	19 (25.7)	16 (24.2)	–
Sex and gender					
Birth-assigned Sex					< 0.001
Male, *n* (%)	99 (44.6)	82 (100.0)^e^	0 (0.0)^f^	17 (25.8)^g^	–
Female, *n* (%)	123 (55.4)	0 (0.0)^e^	74 (100.0)^f^	49 (74.2)^g^	–
Sexual orientation^h^					< 0.001
Heterosexual, *n* (%)	85 (38.3)	46 (56.1)^e^	36 (48.6)^e^	3 (4,5)^f^	–
Non-heterosexual, *n* (%)	137 (61.7)	36 (43,9)^e^	38 (51.4)^e^	63 (95,5)^f^	–
Gender roles					
Bem masculine gender roles, *M* (SE)^i^	4.36 (0.78)	4.45 (0.79)	4.38 (0.73)	4.23 (0.83)	0.228
Bem feminine gender roles, *M* (SE)	5.37 (0.70)	5.22 (0.74)^c^	5.51 (0.67)^b^	5.38 (0.65)	0.033
Bem neutral gender roles, *M* (SE)	4.25 (0,51)	4.37 (0.51)^d^	4.24 (0.51)	4.10 (0.47)^b^	0.005
Storms’ masculinity score, *M* (SE)^j^	2.89 (0.89)	3.60 (0.66)^c,d^	2.15 (0.70)^b,d^	2.85 (0.58)^b,c^	< 0.001
Storms’ femininity score, *M* (SE)	2.82 (0.92)	2.07 (0.63)^c,d^	3.63 (0.64)^b,d^	2.83 (0.66)^b,c^	< 0.001
Gender-affirming and hormonal therapy					< 0.001
Neither, *n* (%)	194 (87.4)	79 (96.3)^e^	71 (95.9)^e^	44 (66.7)^f^	**–**
Hormonal Therapy (HT), *n* (%)	20 (9.0)	3 (3.7)^e^	3 (4.1)^e^	14 (21.2)^f^	**–**
Gender-affirming surgery and HT, *n* (%)	8 (3.6)	0 (0.0)^e^	0 (0.0)^e^	8 (12.1)^f^	**–**
*Behavioral*					
Tobacco smoking					0.699
Smokers, *n* (%)	15 (6.8)	8 (9.8)^e^	4 (5.4)^e^	3 (4.5)^e^	**–**
Social smokers, *n* (%)	44 (19.8)	16 (19.5)^e^	16 (21.6)^e^	12 (18.2)^e^	**–**
Non-smokers, *n* (%)	163 (73.4)	58 (70.7)^e^	54 (73.0)^e^	51 (77.3)^e^	**–**
Alcohol consumption, weekly					0.01
0, *n* (%)	60 (27.0)	20 (24.4)^e,f^	14 (18.9)^f^	26 (39.4)^e^	**–**
1–6, *n* (%)	129 (58.1)	44 (53.7)^e^	49 (66.2)^e^	36 (54.5)^e^	**–**
7 or more, *n* (%)	33 (14.9)	18 (22.0)^e^	11 (14.9)^e,f^	4 (6.1)^f^	**–**
Cannabis consumption					
None, *n* (%)	134 (60.4)	45 (54.9)^e^	56 (75.7)^f^	33 (50.0)^e^	0.004
Occasionally (monthly or annually), *n* (%)	38 (17.1)	18 (22.0)^e^	10 (13.5)^e^	10 (15.2)^e^	**–**
Regularly (daily or weekly), *n* (%)	50 (22.5)	19 (23.2)^e,f^	8 (10.8)^f^	23 (34.8)^e^	**–**
Illicit drug consumption					0.001
None, *n* (%)	187 (84.2)	59 (72.0)^e^	71 (95.9)^f^	57 (86.4)^e,f^	**–**
Occasionally (monthly or annually), *n* (%)	29 (13.1)	18 (22.0)^e^	3 (4.1)^f^	8 (12.1)^e,f^	**–**
Regularly (daily or weekly), *n* (%)	6 (2.7)	5 (6.1)^e^	0 (0.0)^e^	1 (1.5)^e^	**–**
*Contraception and menstruation*					
Postmenopausal, *n* (%)	7 (3.2)	0 (0.0)^e^	2 (2.7)^e,f^	5 (7.6)^f^	0.031
Contraceptive use					< 0.001
None, *n* (%)	180 (81.1)	82 (100.0)^e^	45 (60.8)^f^	53 (80.3)^g^	**–**
Contraceptive pill, *n* (%)	27 (12.2)	0 (0.0)^e^	20 (27.0)^f^	7 (10.6)^g^	**–**
Hormonal IUD, *n* (%)	7 (3.2)	0 (0.0)^e^	3 (4.1)^e^	4 (6.1)^e^	–
Copper IUD, *n* (%)	2 (0.9)	0 (0.0)^e^	2 (2.7)^e^	0 (0.0)^e^	**–**
Other hormonal contraceptives, *n* (%)	6 (2.7)	0 (0.0)^e^	4 (5.4)^e^	2 (3.0)^e^	**–**
General health					
Medication use, *n* (%)	87 (39.2)	23 (28.0)^e^	23 (31.1)^e^	41 (62.1)^f^	0.124
Neurological condition, *n* (%)	13 (5.9)	4 (4.9)^e^	2 (2.7)^e^	7 (10.6)^e^	0.107
Cardiovascular condition, *n* (%)	13 (5.9)	2 (2.4)^e^	4 (5.4)^e^	7 (10.6)^e^	0.034
General condition, *n* (%)	61 (27.5)	19 (23.2)^e^	16 (21.6)^e^	26 (39.4)^e^	< 0.001
Psychiatric history					< 0.001
None, *n* (%)	64 (28.8)	33 (40.2)^e^	25 (33.8)^e^	6 (9.1)^f^	**–**
Past or present history, *n* (%)	32 (14.4)	11 (13.4)^e^	9 (12.2)^e^	12 (18.2)^e^	**–**
Family history, *n* (%)	46 (20.7)	20 (24.4)^e^	19 (25.7)^e^	7 (10.6)^e^	**–**
Both past/present and family history, *n* (%)	79 (35.6)	18 (22.0)^e^	21 (28.4)^e^	40 (60.6)^f^	**–**
Missing, *n* (%)	1 (0.5)	0 (0.0)	0 (0.0)	1 (1.5)	**–**

^a^*M* = mean; SE = standard error

^b^Significantly different from cisgender men after post hoc comparisons using the Tukey HSD test

^c^Significantly different from cisgender women after post hoc comparisons using the Tukey HSD test

^d^Significantly different from gender-diverse people after post hoc comparisons using the Tukey HSD test

^e,f,g^Homogeneous subsets after Chi-squared tests and comparison of column’s proportion; using Bonferroni’s correction

^h^Sexual orientation was assessed using the Kinsey Scale, including 1 (exclusively heterosexual), 2 (predominantly heterosexual, only incidentally homosexual), 3 (predominantly heterosexual, more than incidentally homosexual), 4 (bisexual or pansexual), 5 (predominantly homosexual, more than only incidentally heterosexual), 6 (predominantly homosexual, only incidentally heterosexual), 7 (exclusively homosexual) and 8 (asexual spectrum). If participant identified as 1 or 2, they were classified as heterosexual. If participant identified as 3, 4, 5, 6, 7 or 8, they were classified as non-heterosexual

**Fig. 1:**
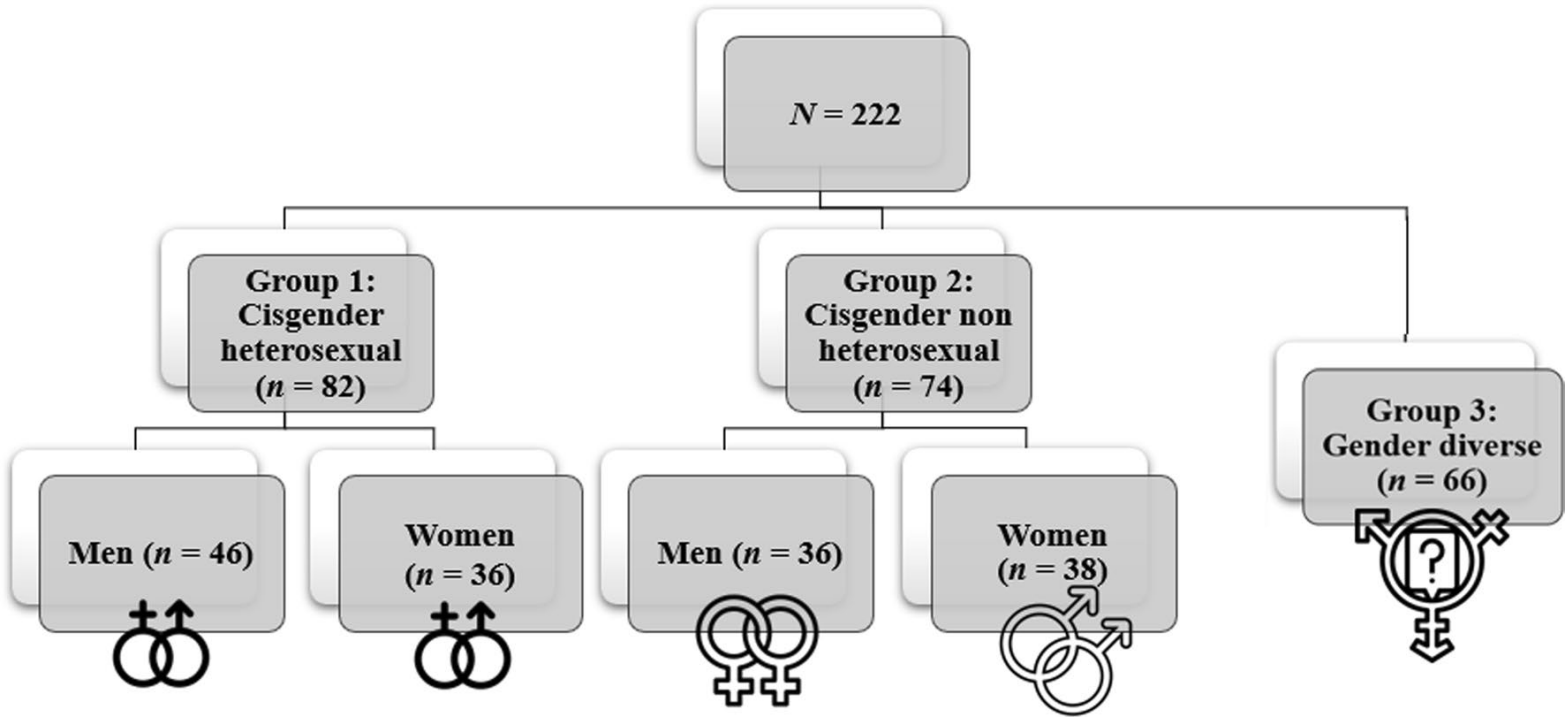
Group and subgroup divisions

Participants (*N* = 222) were divided into three groups and then into five sub-groups (see Fig. 1). The first group (*n* = 82) was divided in two sub-groups, respectively, composed of (1) heterosexual cisgender men (*n* = 46) and (2) heterosexual cisgender women (*n* = 36). The second group was composed of people representing sexual diversity (people who do not identify themselves as only heterosexual) and was divided in two sub-groups: (1) cisgender non-heterosexual men (*n* = 36) and (2) cisgender non-heterosexual women (*n* = 38). The third and last group was composed of people representing gender diversity (e.g., trans men, trans women, non-binary, gender fluid, queers, and others; *n* = 66). These groups have been separated according to the main variables of interest (e.g., birth-assigned sex, gender identity and sexual orientation) in Fig. 1.

### Procedures

**Fig. 2:**
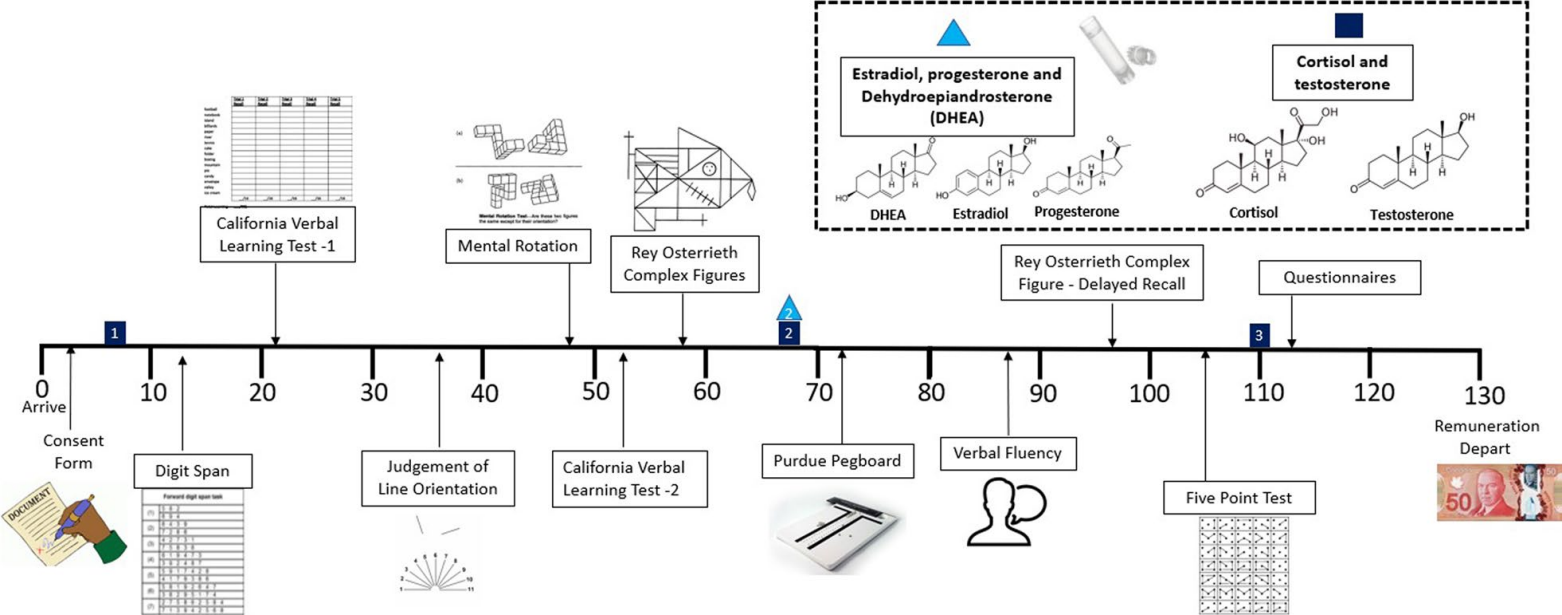
Illustrated protocol

This study is based on a published protocol paper (Kheloui et al., 2021132). Our study was approved by the ethics committee of the Montreal Mental Health University Institute. Interested participants contacted our research team. Following a 10-min telephone screening interview, participants set an appointment at the Center on Sex*Gender, Allostasis, and Resilience (CESAR) based at the Research Center of the Montreal Mental Health University Institute. This study required one visit lasting between 110 to 150 min (*M* = 128.18, SD = 21.30) during which collection of biopsychosocial variables was conducted (see Fig. 2). Visits were scheduled during the afternoon, between 12AM and 5PM to control for circadian variations in basal cortisol (*M* = 14:47 h, SD = 88.16 min).

Participants were provided with all necessary information regarding the protocol at the start of the session. Trained testers reiterated that all data would be kept in complete confidentiality. Upon consent, a first saliva sample was obtained to measure levels of sex hormones, cortisol, and dehydroepiandrosterone. Two more samples were obtained: one after the 5th cognitive task (mid-way) and another after the 8th and last cognitive task. Finally, participants completed a questionnaire on an online platform called Qualtrics. Well-validated questionnaires assessed gender identity, gender roles, and sexual orientation, as well as socioeconomic status, race/ethnicity, menstruation, contraceptive, substance use, medications, and physical and mental health, which can influence performance on cognitive tasks. Participants were compensated $50 (see Fig. 2).

### Measures

#### Biological measures

Participants were asked to produce between 2 to 3 mL of saliva in a tube (Salivettes) assisted with a thick straw. A total of 3 saliva samples were taken at specific time at the beginning, middle, and end of the testing session (see Fig. 2). All three samples were immediately transported into an industrial freezer of our Research Center by our staff where they were kept frozen at − 20 °C until analyses.

Sterilized 3 mL 12.5 × 71 mm screw cap tubes (VWR®, Item No. 10018-762) were used to collect saliva. In preparation for analyses, frozen samples were thawed to room temperature and centrifuged at 1500×*g* for 15 min. High-sensitivity enzymeimmunoassays was used for cortisol (Salimetrics®, No. 1-3002, sensitivity: 0.012–3 μg/dl), estradiol (Salimetrics®, No. 1-3702, sensitivity: 1–32 pg/mL), progesterone (Salimetrics®, No. 1-1502, sensitivity: 5 pg/mL) and DHEA (Salimetrics®, No. 1-1202, sensitivity: 5 pg/mL). Testosterone was determined by expanded-range enzymeimmune assay (Salimetrics®, No. 1-2402, sensitivity: 1 pg/mL). Inter- and intra-assay coefficients of variance were determined for all 5 hormones. Assays will then be duplicated and averaged.

Since cortisol and testosterone showed decrements in concentrations throughout the completion of the protocol in preliminary analyses, we measured only these two hormones at each of the three time-measures to assess dynamics. However, for estradiol, progesterone, and DHEA, only the second saliva sample, taken shortly after the first hour, was included. This decision was made following preliminary analyses with the first ten participants, for whom time effects were observed for testosterone and cortisol, but not for the other biomarkers of interest.

Even though this study does not constitute a stress paradigm, circulating cortisol and DHEA concentrations can impact cognitive abilities [132, 133]. Moreover, cortisol activity can influence sex hormone secretion and should be considered in studies aiming to better understanding sex hormone effects on cognition [86]. For the purpose of main analyses, cortisol and DHEA were combined. The ratio between circulating cortisol and DHEA is considered more accurate and physiological reflection of net cortisol activity [134].

#### Biological confounders of sex hormones

Several potential confounding variables of our biological measures were also considered. Indeed, hormonal contraceptive use was ascertained as well as the presence of hormonal therapy. For analysis purposes, presence of hormonal contraceptive was indexed as a dichotomic variable (0 = absence of hormonal contraceptive, 1 = presence of hormonal contraceptive). Hormonal therapy history, on the other hand, was indexed as a ordinal variable (0 = no hormonal therapy, 1 = hormonal therapy, 2 = gender-affirming surgery & hormonal therapy). Finally, the list of medications taken was requested to control for prescriptions that could modify the secretion and synthesis of sex hormones (see Physical and mental health).

#### Cognitive measures

Performance on the cognitive tasks presented next are the main dependent variables for this study. This battery of cognitive tests covers several neuropsychological functions, for which the majority present a sexual polymorphism in their respective performance according to scientific literature [131]. Cognitive tasks were coded by trained raters that were not blind to participants’ sex or gender profile since raters were often also testers. To constrain multiple comparisons given the eight tasks assessed, inter-correlated scores were averaged for certain tasks as described below. Among the eight tasks that composed this battery, three of them showed better performance for men while three others showed better performance for women. The two remaining tasks presented no significant sex difference in performance.

##### “Male/men-typed” tasks

Mental rotation skills were measured using the Shepard and Meltzer Mental Rotation task [135]. Twenty pairs of objects were presented, all composed of three-dimensionally drawn blocks, to which the participants had to mentally rotate and indicate if they were the same or different. Scores could range from 0 to 20 and participants were given a 3-min limit. The reaction time of each item was reported as additional data. Sex differences have been well documented [136–138] in mental rotation with men outperforming women.

Visuospatial judgement was measured using the 30-item Benton Judgement of Line Orientation task (JLO) [47]. Participants were given a booklet containing 5 practice-items, followed by 30 test-items. Each item consisted of two unnumbered angled lines. The task was to indicate the two numbers that matched the 11 numbered lines of a reference card. Scores could range from 0 to 30. Better performance has been observed among men in comparison to women [48, 49].

The Rey–Osterrieth Complex Figure test (ROCF) measures spatial memory alongside visuospatial constructional ability [139, 140]. While sex differences have been reported in this task (where men outperform women), some studies reported low effect sizes [141, 142]. The task was carried out in three phases, starting with the copy of the figure, without a time limit. Once completed, the experimenter left the room, leaving the participant alone for 3 min. The second phase began as soon as the experimenter returned, where the figure had to be redrawn from memory, without a time limit. The last phase of the task occurred later in the protocol, about 40 min after the second, during which the figure was redrawn a second and last time from memory. Scores varied from 0 to 36 (between 0 and 2 points were allocated for each 18 items, based on exactitude and location). Immediate and delayed recall scores are frequently used together to observe consolidation in long-term memory [143, 144]. Nevertheless, given the strong correlation between those two measures (*r* = 0.960), we averaged both scores (see Additional file 1).

##### “Female/women-typed” tasks

Verbal memory is a cognitive domain for which sex differences have been observed [35]. This neuropsychological function was measured using the California Verbal Learning Test Second-Edition (CVLT-II) [145]. Studies underline sex differences in CVLT, where women’s performance is generally better than men’s [29, 146, 147]. The completion of this task took about 15 min and took place in two phases. Participants were asked to memorize and recall a first list of 16 words read out loud by the experimenter. This short sequence was repeated five times for the same list, giving a score ranging from 0 to 80 (5 list-recall of 16 words each). This section was followed by a similar exercise of memorizing and recalling a second list of 16 words, giving a score from 0 to 16. Participants were asked to continue this task by listing the most words of the first list as they remembered directly after recalling the second list, and another time 30 min after, each getting a score from 0 to 16. For simplicity, the measures used for analyses were only the sum of trial 1 to 5 (ranging from 0 to 80). This decision was based on the followed premise: this measure is the most reported measure in studies using the California Verbal Learning Test and it provides a reliable index of verbal learning and verbal memory [148, 149].

Semantic verbal fluency was measured using the Controlled Word Association task [150]. Participants were asked to generate as many words as they could from a certain category. Animals, fruits, and vegetables were the ones chosen and 1 min was the time allowed for each of these. Scores of this task were determined by adding the total of correct words generated by all three categories. Studies have shown that women outperform men in verbal fluency [151].

The Purdue Pegboard task measures motor skills [152]. Sex differences have been observed using this task with women performing better than men [153, 154]. The material for this task consists of a board with two parallel rows of 25 equidistant holes and several dozen pieces of three types. The task involves performing four different manipulations with these pieces: one only with the right hand, one with the left hand, one with both hands at the same time, and one where the three pieces were alternated to form an assembly. This cycle of four manipulations was executed three times. Scores of the first three manipulations were defined by the number of pieces placed into the board after 30 s. The sum of these three manipulations was calculated and averaged for the three trials. Assembly scores were determined by multiplying by four the number of complete structures built, over a maximum of 60 s. These scores were also averaged around the three trials. In the same way as the Rey–Osterrieth Complex Figure, two scores were generated from this task. Given the high correlation between these two scores (*r* = 0.577), we averaged both scores (see Additional file 1).

##### “Neutral-typed” tasks

The two last tasks incorporated in the protocol either showed no significant sex difference or showed a sexual polymorphism that researchers considered too inconsistent across different studies [155–158]. The addition of these tasks in a protocol was as control conditions with tasks where no sex differences was expected.

The Digit Span task was chosen as the first “ice-breaker” task between participants and testers [155]. Immediate memory was the neuropsychological function measured that takes approximately 10 min to complete. People were asked to recall the sequence of numbers named by the experimenter in the correct order. The task started with lists of two digits and progressed to lists of ten digits. The score on this task was summarized by the number of digits in the longest successful sequence.

The Five-Point Test was the last task of this protocol and measured figural fluency functions [159]. No sex differences were found even after many studies developing norms on numerous subpopulations [157, 158, 160]. Performance varies significantly according to education level and age [161]. Completion of this task lasts 2 min and is done on a page with 35 identical squares with 5 dots. Participants had to make as many unique drawings as possible, using only straight lines. Scores were integers from 0 to 35, according to the number of correct and unique drawings.

#### Sociodemographic and psychosocial measures

##### Birth-assigned sex and gender identity

Birth-assigned sex and gender identity were measured using an adapted version of a scale developed by Bauer [162]. This questionnaire measures birth-assigned sex and gender identity both with one item. The gender identity item assessed the gender identity that the person identifies with the most.

##### Characteristic gender roles

Gender role were addressed using the Bem Sex Role Inventory—Short Form [163, 164]. This questionnaire presented 30 gender-stereotyped traits to which participants were asked to assess the level at which they were embodied on a 7-point Likert scale (1 = never or almost never true, to 7 = always or almost always true). 10 items were, respectively, considered masculine and feminine, alongside 10 items that were considered neutral and which measure social desirability. Two scores for this questionnaire are calculated and determined by the means of the 10 masculine items and the 10 feminine items. This short version of the BSRI presented a 0.90 correlation with its original version, published 7 years earlier [164, 165]. Internal consistency for this scale was measured for the 10 masculine/feminine items for each of our three gender identity groups: cisgender men, cisgender women and gender diverse. Masculinity showed acceptable Cronbach alpha’s (cisgender men: α = 0.79; cisgender women: α = 0.76; gender diverse: α = 0.79). Similarly, femininity showed sufficient Cronbach alpha’s (cisgender men: α = 0.79; cisgender women: α = 0.84; gender diverse: α = 0.74).

##### Sexual orientation

Sexual orientation was assessed using a modified Kinsey scale [166]. This classic scale provides a dimensional measure over and above homosexuality–heterosexuality categorical responses. The scale includes measures ranging from 0 (exclusively heterosexual) to 6 (exclusively homosexual). In addition to these seven measures, we have added a score (7) to the scale, including along people identifying on the asexuality spectrum. Moreover, given that pansexuality was not considered in the original scale, pansexual individuals were attributed the same score as bisexual individuals. This type of measure will allow analyses on a dimensional level (exclusively heterosexual vs exclusively homosexual) and on a categorical level (heterosexual and non-heterosexual). Scores from 0 to 1 will form the "heterosexual" category, while scores from 2 to 7 will form the "non-heterosexual" one.

##### Drug and alcohol use

A three-item short screening questionnaire was designed to measure alcohol, illicit drug (i.e., cocaine, ecstasy, amphetamines) and cannabis consumption. The average number of alcoholic beverages consumed per week was asked. Three levels were defined: (1) no alcohol consumption, (2) between one and six beverages a week, and (3) seven or more beverages a week. These categories were chosen according to the Canadian Guidelines for alcohol use disorder [167]. Similarly, the profile of illicit drug consumption was assessed through a three-level scale: (1) no illicit drug consumption, (2) monthly or annually consumption, and (3) daily or weekly consumption. Finally, cannabis consumption was defined with the same three-level scale as used for the illicit drug consumption. For statistical analysis purposes, the three consumption behaviors were combined and indexed as follow: the sum of the scores of the three scales (alcohol, cannabis, and illicit drugs), each having three levels, ranging from 0 (no consumption) to 2 (regular consumption). This said, score for this index went from 0 to 9.

##### Physical and mental health

Physical general health was assessed with a screening questionnaire. Participants had to indicate which medical illness from the conditions listed applied to their profile in a three-part questionnaire: cardiovascular conditions (e.g., heart attack, hypo/hypertension, and more), neurological conditions (e.g., stroke, epilepsy, and more) and general conditions (e.g., diabetes, sexually transmitted diseases, and more).

Furthermore, mental health was assessed according to different psychiatric condition (e.g., depression, bipolar disorder, schizophrenia, and more), to which they indicated if the diagnosis applied to their profile. The same questions were asked again for immediate family members (mother, father, brother, sister). Following a similar indexing manner as the one for substance use, a physical and mental health index was created. This one went from 0 to 5 and was the sum of 5 dichotomic scores (if the participant took medication, had a neurological, cardiovascular, or general health condition, and had a psychiatric history).

### Statistical analyses

Statistical analyses were performed using the Statistical Package for the Social Sciences (SPSS) Version 28 software. An a priori power analysis was conducted using G*Power version 3.1.9.4 to determine the minimum sample size required to perform linear hierarchical multiple regressions, with a *R*^2^ increase using the Enter method. To detect sex/gender effects using 9 factors and 5 covariates, while explaining 10% of the variance [168] at 80% power, at a significance criterion of α = 0.05, a sample size of *N* = 196 was needed. An expected effect size of 0.10 was determined based on effect sizes reported in recent SPC articles. With an addition of two a posteriori covariates, a minimum sample size of *N* = 207 was required. Our final sample size of *N* = 222 was therefore adequate.

Preliminary analyses assessed demographics, substance use and general physical and mental health using analyses of variance (ANOVA) or χ^2^ (according to the categorical or continuous nature of each variable), as a function of gender identity (see Table 1). Post hoc analyses used Tukey’s test. Two correlation matrices using Pearson correlations were produced: one to describe multiple associations between cognitive tasks (Additional file 1: Table S1), and one to describe multiple associations between sex and gender variables of interest (Table 2).

**Table 2:** Correlation matrix of sex*gender variables

Variables	1	2	3	4	5	6	7	8
1. Birth-assigned sex^a^	–							
2. Testosterone	− 0.504^***^	–						
3. Estradiol	0.235^***^	0.012	–					
4. Progesterone	0.333^***^	− 0.046	0.463^***^	–				
5. Gender identity, woman^b^	0.597^***^	− 0.498^***^	0.198^**^	0.151^*^	–			
6. Gender identity, GD^b^	0.221^***^	− 0.022	0.058	0.124^†^	− 0.418^***^	–		
7. BEM masculine	− 0.097	− 0.010	− 0.188^**^	− 0.111^†^	− 0.012	− 0.031	–	
8. BEM feminine	0.160*	− 0.095	0.186^**^	0.055	0.147^*^	0.036	0.065	–
9. Sexual orientation^c^	0.207^**^	− 0.004	0.031	0.045	− 0.146^*^	0.409^***^	− 0.088	0.053

^†^*p* < 0.10; *p < 0.05; **p < 0.01; ***p < 0.001

^a^Sex was added as a dummy-coded variable (0 = "Male", 1 = "Female")

^b^For gender identity, "men" was chosen as reference group and compared to "women" (contrast Gender Identity 1) and "non-binary or a similar identity" (contrast Gender Identity 2)

Main analyses were organized in two parts according to our two hypotheses. First, analyses of variance (ANOVA) were conducted to ascertain birth-assigned sex differences for the eight cognitive tasks. Significance was set at α = 0.05 and effect sizes are reported as partial eta squared (η^2^_P_). Effect sizes can be interpreted as a small effect (η^2^_P_ ≅ 0.01), medium effect (η^2^_P_ ≅ 0.06), or large effect (η^2^_P_ ≅ 0.14) [169]. To more easily allow comparisons in the discussion, we have provided an interpretation scale for Cohen's D, because SPC literature mainly uses this measure of effect size. Cohen’s d can be interpreted as a small effect (*d* ≅ 0.2), medium effect (*d* ≅ 0.5), or large effect (*d* ≅ 0.8) [169]. Second, multiple hierarchical regressions were performed, with the aim of including the five sex and gender factors previously mentioned in sequence. Hierarchical blocks were added using the Enter method and were designed as followed: (1) birth-assigned sex (coded as male = 0 and female = 1); (2) sex hormones (testosterone, estradiol and progesterone); (3) gender identity dummy variables as women (coded women as referent = 0 and those that do not identify as a women = 1) and as gender diverse (coded gender diverse as referent = 0 and cisgender = 1); (4) gender roles (masculinity and femininity scale); (5) sexual orientation (coded as heterosexual = 0 and non-heterosexual = 1); and finally (6) covariates added last. We justified adding covariates in the last block (as opposed to the first block) so as to allow assessment of unadjusted associations specific to sex, gender, and sexual orientation in sequence.

Covariates were selected a priori based on the literature showing that age [170, 171], language [172], hormone-replacement therapy [173, 174], DHEA/cortisol ratio [175], and contraceptive use [176] have impacts on the cognitive abilities. Based on preliminary analyses of group differences, further a posteriori covariates were selected. These included alcohol and drug use [177, 178] and physical and mental health conditions [179, 180]. ANOVA and regressions’ assumption of score independence, normality, linearity, and homoscedasticity were respected following recommendations [181]. Independent variables also met assumptions of collinearity according to variance inflation factor (VIF) test: VIF [1.079, 3.708] [182]. Each variable included in this model is presented in Table 2.

## Results

### Preliminary results

Table 1 shows sample’s descriptive statistics as a function of gender identity. Men were significantly older than women (*p* = 0.047). Group differences according to mother tongue were also observed, where the proportion of English speakers was higher for gender-diverse people than for other groups (*p* = 0.022). People who were undergoing hormonal therapy or had received gender-affirming surgery were significantly more represented in the gender-diverse group (*p* < 0.001). The gender-diverse group were also using more medications (*p* < 0.001), presented higher past or present psychiatric conditions (*p* < 0.001), and had more health conditions (*p* = 0.034). No group differences were observed for cardiovascular or neurological conditions. Men had significantly higher alcohol consumption (*p* = 0.010) and illicit drug use (*p* = 0.001) compared to women and gender-diverse people. Men and gender-diverse people also consumed more cannabis than women (*p* = 0.004). No group differences in years of education or race/ethnicity were observed. Additional file 1: Tables S1 and S2 are correlation matrices of key study variables reported for descriptive purposes.

### Main analyses

#### Cognitive performances in relation to birth-assigned sex

**Fig. 3:**
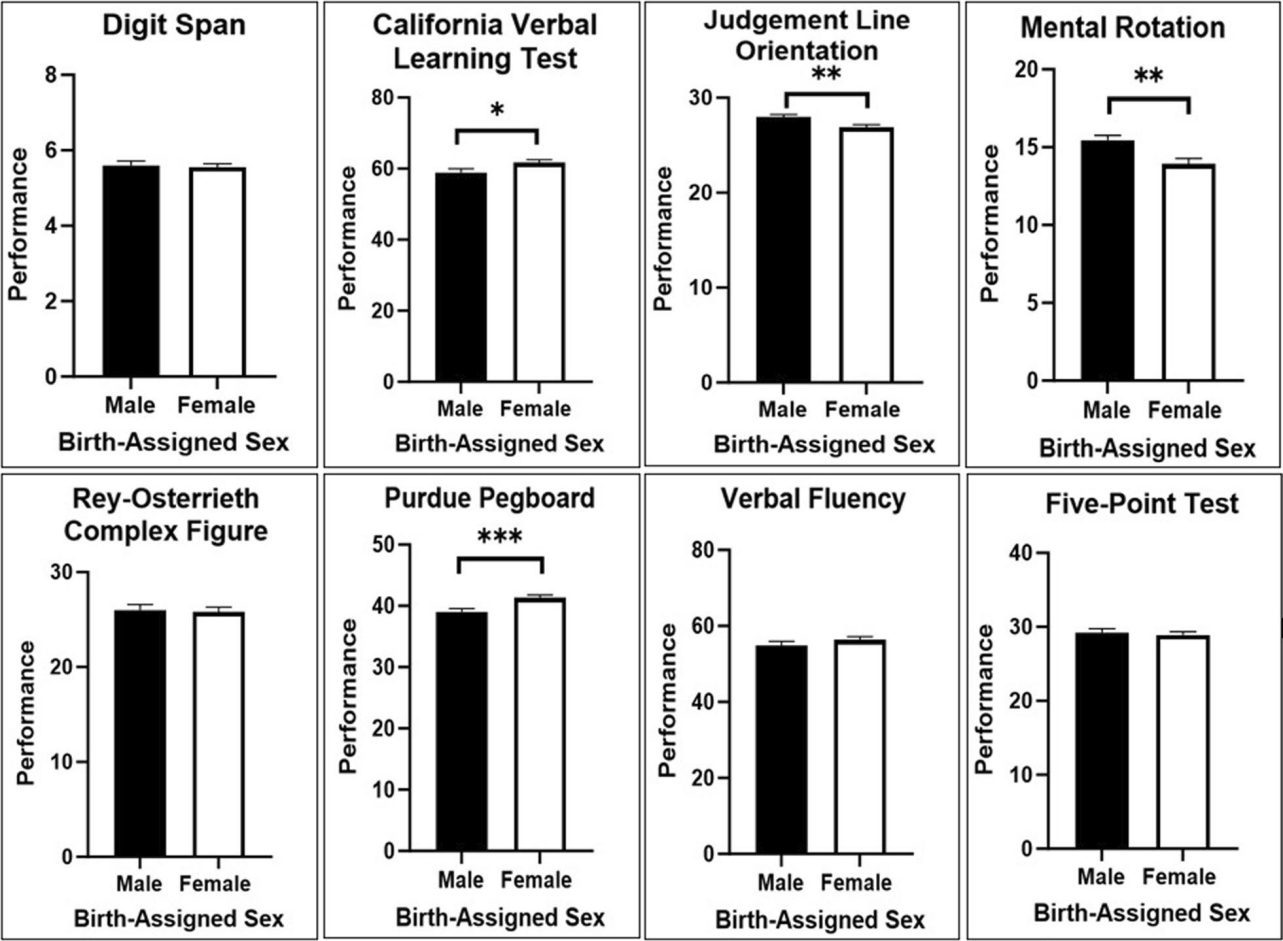
Cognitive performances in relation to birth-assigned sex. *p < 0.05; **p < 0.01; ***p < 0.001

One-way ANOVAs were conducted to determine birth-assigned sex differences for all eight cognitive tasks (see Fig. 3 and Table 3). Results revealed that males performed significantly better than females on the Mental Rotation [*F*_(1,220)_ = 10.069, *p* = 0.002, η^2^ = 0.044] and in Judgement Line Orientation [*F*_(1,220)_ = 7.331, *p* = 0.007, η^2^ = 0.032]. By contrast, females performed significantly better than males on the California Verbal Learning Test [*F*_(1,220)_ = 4.429, *p* = 0.036, η^2^ = 0.020] and the Purdue Pegboard test [*F*_(1,218)_ = 11. 312, *p* < 0.001, η^2^ = 0.049’]. No significant differences were observed between males and females in Digit Span [*F*_(1,220)_ = 0.147, *p* = 0.701, η^2^ = 0.001], in Rey–Osterrieth Complex Figure, [*F*_(1,220)_ = 0.081, *p* = 0.777, η^2^ = 0.000], in Verbal Fluency, [*F*_(1,219)_ = 1.040, *p* = 0.309, η^2^ = 0.005], and in Five-Point Test [*F*_(1,220)_ = 0.298, *p* = 0.586, η^2^ = 0.001].

**Table 3:** Cognitive performance in relation to birth-assigned sex

Cognitive measures	Male		Female		*F*_(1, 220)_	η^2^_P_
	*M*	SD	*M*	SD		
Digit Span	5.61	1.09	5.55	1.04	0.147	0.001
California Verbal Learning Test	58.92	10.22	61.68	9.31	4.429^*^	0.020
Judgement Line Orientation	27.96	2.61	26.87	3.25	7.331^**^	0.032
Mental Rotation	15.46	2.86	13.90	4.17	10.069^**^	0.044
Rey–Osterrieth Complex Figure	26.01	5.61	25.80	5.73	0.081	0.000
Purdue Pegboard	39.05	5.01	41.32	4.95	11.312^***^	0.049
Verbal Fluency	54.81	11.37	56.27	9.94	1.040	0.005
Five-Point Test	29.28	4.99	28.92	4.91	0.298	0.001

*p < 0.05; **p < 0.01; ***p < 0.001

#### Cognitive performance according to the five sex and gender factors

As described earlier, hierarchical regressions used six blocks to predict cognitive performance (Tables 4, 5, 6, 7, 8, 9, and Additional file 1: Tables S2 and S3). Model 1 predicted cognitive performance according to birth-assigned sex; Model 2 added sex hormones, and Model 3, 4 and 5 added, respectively, gender identity, gender roles and sexual orientation. Model 6 included our seven covariates. Table 2 reports a correlation matrix of key study variables reported for descriptive purposes.

**Table 4:** Regression table of California verbal learning test performances according to all five sex*gender factors and covariates

Predictor factors	*R*^2^	*R*^2^ adjusted	Δ*R*^2^	*p*	*B*	SE B	β	*t*	*p*	95% CI: LL	95% CI: UL
Model 1	0.012	0.007	0.012	0.116							
Birth-assigned sex (male = 0, female = 1)					2.152	1.362	0.109	1.580	0.116	− 0.533	4.837
Model 2	0.045	0.026	0.033	0.050							
Testosterone					0.019	0.011	0.141	1.762	0.080	− 0.002	0.040
Estradiol					− 3.186	1.553	− 0.157	− 2.052	0.041	− 6.247	− 0.124
Progesterone					0.012	0.009	0.098	1.249	0.213	− 0.007	0.030
Model 3	0.066	0.039	0.021	0.029							
Gender identity, woman (not a woman: 0, woman: 1)					− 1.192	2.642	− 0.057	− 0.451	0.653	− 6.402	4.018
Gender identity, GD (not GD = 0, GD = 1)					2.581	2.261	0.115	1.142	0.255	− 1.876	7.038
Model 4	0.078	0.041	0.012	0.035							
BEM femininity					− 1.208	1.001	− 0.086	− 1.207	0.229	− 3.183	0.766
BEM masculinity					− 0.811	0.892	− 0.064	− 0.909	0.365	− 2.570	0.949
Model 5	0.095	0.055	0.017	0.016							
Sexual orientation (0 = heterosexual, 1 = non-heterosexual)					2.999	1.532	0.148	1.958	0.052	− 0.021	6.019
Model 6: covariates	0.136	0.064	0.041	0.023							
Age					− 0.193	0.086	− 0.168	− 2.240	0.026	− 0.362	− 0.023
DHEA/cortisol ratio					< 0.001	0.001	− 0.006	− 0.086	0.932	− 0.002	0.002
Language (0 = French, 1 = English)					− 1.494	2.025	− 0.054	− 0.738	0.462	− 5.488	2.500
Hormone and surgery index					1.736	1.699	0.081	1.022	0.308	− 1.615	5.087
Contraceptive use (0 = no contraceptive, 1 = contraceptive)					0.928	1.962	0.037	0.473	0.637	− 2.942	4.797
Substance use index					0.841	0.534	0.111	1.577	0.116	− 0.211	1.894
Physical and mental health index					0.488	0.931	0.038	0.525	0.601	− 1.348	2.324

**Table 5:** Regression table of judgement line orientation performances according to all five sex*gender factors and covariates

Predictor factors	*R*^2^	*R*^2^ adjusted	Δ*R*^2^	*p*	*B*	SE B	β	*t*	*p*	95% CI: LL	95% CI: UL
Model 1	0.035	0.030	0.035	0.007							
Birth-assigned sex (male = 0, female = 1)					− 1.120	0.409	− 0.187	− 2.740	0.007	− 1.926	− 0.314
Model 2	0.074	0.056	0.039	0.003							
Testosterone					0.005	0.003	0.116	1.467	0.144	− 0.002	0.011
Estradiol					− 0.958	0.464	− 0.156	− 2.064	0.040	− 1.874	− 0.043
Progesterone					0.006	0.003	0.167	2.161	0.032	0.001	0.011
Model 3	0.104	0.078	0.030	< 0.001							
Gender identity, woman (not a woman: 0, woman: 1)					− 0.832	0.786	− 0.132	− 1.059	0.291	− 2.382	0.718
Gender identity, GD (Not GD = 0, GD = 1)					0.607	0.672	0.089	0.903	0.368	− 0.719	1.933
Model 4	0.108	0.073	0.004	0.003							
BEM femininity					0.159	0.299	0.037	0.532	0.595	− 0.430	0.749
BEM masculinity					− 0.237	0.266	− 0.061	− 0.891	0.374	− 0.763	0.288
Model 5	0.118	0.078	0.009	0.002							
Sexual orientation (0 = heterosexual, 1 = non-heterosexual)					0.670	0.459	0.109	1.458	0.146	− 0.236	1.576
Model 6: covariates	0.135	0.063	0.017	0.024							
Age					− 0.016	0.026	− 0.046	− 0.614	0.540	− 0.068	0.036
DHEA/cortisol ratio					0.000	0.000	− 0.079	− 1.109	0.269	− 0.001	0.000
Language (0 = French, 1 = English)					0.173	0.615	0.021	0.282	0.778	− 1.040	1.387
Hormone and surgery index					0.535	0.516	0.083	1.037	0.301	− 0.483	1.554
Contraceptive use (0 = no contraceptive, 1 = contraceptive)					0.340	0.596	0.045	0.571	0.569	− 0.836	1.516
Substance use index					0.136	0.162	0.059	0.842	0.401	− 0.183	0.456
Physical and mental health index					0.053	0.283	0.014	0.189	0.850	− 0.504	0.611

**Table 6:** Regression table of mental rotation performances according to all five sex*gender factors and covariates

Predictor factors	*R*^2^	*R*^2^ adjusted	Δ*R*^2^	*p*	*B*	SE B	β	*t*	*p*	95% CI: LL	95% CI: UL
Model 1	0.042	0.038	0.042	0.003							
Birth-assigned sex (male = 0, female = 1)					− 1.530	0.504	− 0.206	− 3.038	0.003	− 2.523	− 0.537
Model 2	0.044	0.026	0.002	0.054							
Testosterone					− 0.001	0.004	− 0.029	− 0.355	0.723	− 0.009	0.007
Estradiol					− 0.190	0.584	− 0.025	− 0.325	0.746	− 1.340	0.961
Progesterone					0.002	0.003	0.037	0.473	0.637	− 0.005	0.009
Model 3	0.045	0.017	0.001	0.148							
Gender identity, woman (not a woman: 0, woman: 1)					− 0.468	1.004	− 0.060	− 0.467	0.641	− 2.447	1.510
Gender identity, GD (Not GD = 0, GD = 1)					− 0.258	0.859	− 0.031	− 0.300	0.764	− 1.951	1.435
Model 4	0.056	0.019	0.011	0.159							
BEM femininity					0.105	0.380	0.020	0.277	0.782	− 0.645	0.856
BEM masculinity					− 0.521	0.339	− 0.109	− 1.538	0.126	− 1.190	0.147
Model 5	0.062	0.020	0.006	0.162							
Sexual orientation (0 = heterosexual, 1 = non-heterosexual)					0.639	0.586	0.084	1.091	0.277	− 0.516	1.794
Model 6: covariates	0.113	0.039	0.051	0.091							
Age					− 0.053	0.033	− 0.122	− 1.613	0.108	− 0.117	0.012
DHEA/cortisol ratio					0.000	0.000	− 0.078	− 1.090	0.277	− 0.001	0.000
Language (0 = French, 1 = English)					− 0.932	0.771	− 0.089	− 1.209	0.228	− 2.452	0.588
Hormone and surgery index					1.048	0.647	0.131	1.620	0.107	− 0.228	2.323
Contraceptive use (0 = no contraceptive, 1 = contraceptive)					0.523	0.747	0.056	0.700	0.485	− 0.950	1.995
Substance use index					0.045	0.203	0.016	0.223	0.824	− 0.355	0.446
Physical and mental health index					− 0.673	0.354	− 0.139	− 1.899	0.059	− 1.372	0.026

**Table 7:** Regression table of Rey–Osterrieth Complex Figure performances according to all five sex*gender factors and covariates

Predictor factors	*R*^2^	*r*^2^ adjusted	Δ*R*^2^	*p*	*B*	SE B	β	*t*	*p*	95% CI: LL	95% CI: UL
Model 1	0.000	− 0.005	0.000	0.850							
Birth-assigned sex (male = 0, female = 1)					− 0.148	0.781	− 0.013	− 0.190	0.850	− 1.689	1.392
Model 2	0.012	− 0.008	0.012	0.657							
Testosterone					0.008	0.006	0.101	1.239	0.217	− 0.005	0.020
Estradiol					− 0.966	0.901	− 0.084	− 1.072	0.285	− 2.742	0.810
Progesterone					0.001	0.005	0.016	0.200	0.842	− 0.010	0.012
Model 3	0.029	0.000	0.017	0.430							
Gender identity, woman (not a woman: 0, woman: 1)					− 1.842	1.537	− 0.155	− 1.198	0.232	− 4.873	1.189
Gender identity, GD (not GD = 0, GD = 1)					0.194	1.315	0.015	0.148	0.883	− 2.399	2.787
Model 4	0.031	− 0.007	0.003	0.596							
BEM femininity					0.380	0.585	0.047	0.649	0.517	− 0.774	1.534
BEM masculinity					0.141	0.522	0.019	0.270	0.788	− 0.888	1.169
Model 5	0.037	− 0.006	0.006	0.567							
Sexual orientation (0 = heterosexual, 1 = non-heterosexual)					0.995	0.901	0.086	1.104	0.271	− 0.782	2.772
Model 6: covariates	0.104	0.029	0.067	0.148							
Age					− 0.135	0.050	− 0.206	− 2.700	0.008	− 0.234	− 0.036
DHEA/cortisol ratio					0.000	0.000	0.040	0.550	0.583	− 0.001	0.001
Language (0 = French, 1 = English)					− 1.685	1.176	− 0.107	− 1.433	0.154	− 4.005	0.635
Hormone and surgery index					0.625	0.987	0.051	0.633	0.528	− 1.322	2.571
Contraceptive use (0 = no contraceptive, 1 = contraceptive)					0.368	1.140	0.026	0.323	0.747	− 1.879	2.616
Substance use index					0.531	0.310	0.123	1.715	0.088	− 0.080	1.143
Physical and mental health index					0.690	0.541	0.094	1.276	0.204	− 0.377	1.756

**Table 8:** Regression table of Purdue Pegboard performances according to all five sex*gender factors and covariates

Predictor factors	*R*^2^	*R*^2^ adjusted	Δ*R*^2^	*p*	*B*	SE B	β	*t*	*p*	95% CI: LL	95% CI: UL
Model 1	0.046	0.042	0.046	0.002							
Birth-assigned sex (male = 0, female = 1)					2.166	0.685	0.215	3.163	0.002	0.816	3.515
Model 2	0.059	0.040	0.013	0.015							
Testosterone					0.003	0.005	0.043	0.536	0.593	− 0.008	0.014
Estradiol					− 1.124	0.799	− 0.108	− 1.406	0.161	− 2.701	0.452
Progesterone					0.006	0.005	0.094	1.205	0.230	− 0.004	0.015
Model 3	0.066	0.038	0.007	0.032							
Gender identity, woman (not a woman: 0, woman: 1)					0.062	1.349	0.006	0.046	0.963	− 2.597	2.721
Gender identity, GD (not GD = 0, GD = 1)					1.011	1.154	0.088	0.876	0.382	− 1.264	3.286
Model 4	0.070	0.032	0.004	0.067							
BEM femininity					− 0.405	0.513	− 0.056	− 0.790	0.431	− 1.417	0.607
BEM masculinity					0.270	0.458	0.042	0.590	0.556	− 0.633	1.173
Model 5	0.070	0.028	0.000	0.103							
Sexual orientation (0 = heterosexual, 1 = non-heterosexual)					− 0.030	0.798	− 0.003	− 0.037	0.970	− 1.603	1.544
Model 6: covariates	0.137	0.064	0.067	0.023							
Age					− 0.142	0.044	− 0.239	− 3.210	0.002	− 0.229	− 0.055
DHEA/cortisol ratio					0.000	0.000	− 0.039	− 0.543	0.588	− 0.001	0.001
Language (0 = French, 1 = English)					− 0.279	1.031	− 0.020	− 0.271	0.787	− 2.313	1.755
Hormone and surgery index					0.457	0.866	0.042	0.527	0.599	− 1.252	2.166
Contraceptive use (0 = no contraceptive, 1 = contraceptive)					1.365	1.002	0.108	1.362	0.175	− 0.612	3.343
Substance use index					0.313	0.272	0.082	1.151	0.251	− 0.224	0.850
Physical and mental health index					0.372	0.474	0.057	0.784	0.434	− 0.564	1.308

**Table 9:** Regression table of verbal fluency performances according to all five sex*gender factors and covariates

Predictor factors	*R*^2^	*R*^2^ adjusted	Δ*R*^2^	*p*	*B*	SE B	β	*t*	*p*	95% CI: LL	95% CI: UL
Model 1	0.006	0.001	0.006	0.280							
Birth-assigned sex (male = 0, female = 1)					1.589	1.468	0.075	1.083	0.280	− 1.305	4.484
Model 2	0.008	− 0.011	0.002	0.802							
Testosterone					0.000	0.012	− 0.001	− 0.009	0.993	− 0.023	0.023
Estradiol					− 0.412	1.697	− 0.019	− 0.243	0.808	− 3.759	2.934
Progesterone					− 0.005	0.010	− 0.040	− 0.498	0.619	− 0.025	0.015
Model 3	0.014	− 0.016	0.006	0.830							
Gender identity, woman (not a woman: 0, woman: 1)					− 0.460	2.926	− 0.021	− 0.157	0.875	− 6.229	5.309
Gender identity, GD (Not GD = 0, GD = 1)					1.598	2.492	0.066	0.641	0.522	− 3.316	6.511
Model 4	0.018	− 0.021	0.004	0.887							
BEM femininity					0.621	1.109	0.041	0.560	0.576	− 1.565	2.808
BEM masculinity					− 0.778	0.990	− 0.057	− 0.786	0.433	− 2.730	1.175
Model 5	0.018	− 0.026	0.000	0.929							
Sexual orientation (0 = heterosexual, 1 = non-heterosexual)					0.443	1.714	0.020	0.259	0.796	− 2.937	3.824
Model 6: covariates	0.084	0.008	0.066	0.358							
Age					0.051	0.095	0.042	0.540	0.590	− 0.136	0.239
DHEA/cortisol ratio					− 0.001	0.001	− 0.060	− 0.826	0.410	− 0.003	0.001
Language (0 = French, 1 = English)					− 3.710	2.237	− 0.125	− 1.659	0.099	− 8.122	0.702
Hormone and surgery index					1.032	1.888	0.045	0.547	0.585	− 2.691	4.755
Contraceptive use (0 = no contraceptive, 1 = contraceptive)					− 2.096	2.184	− 0.078	− 0.959	0.339	− 6.405	2.213
Substance use index					1.504	0.590	0.186	2.548	0.012	0.340	2.668
Physical and mental health index					1.922	1.030	0.139	1.866	0.064	− 0.109	3.954

##### Verbal cognitive tasks

The model predicting the California Verbal Learning Test performance according to birth-assigned sex accounted for a non-significant variance [*F*_(1, 208)_ = 2.496, *p* = 0.116, *R*^2^ = 0.012]. The addition of sex hormones improved the model, as Model 2 became significant [*F*_(4, 205)_ = 2.414, *p* = 0.050, *R*^2^ = 0.045]. Specifically, lower estradiol levels were associated with higher performance (*p* = 0.041). Adding gender identity to Model 3 increased variance explained, and remained significant [*F*_(6, 203)_ = 2.398, *p* = 0.029, *R*^2^ = 0.066]. This variable was separated into two sub-variables for the purpose of analyses (women vs non-women and cisgender vs non-cisgender). However, when we investigated effects of each sub-variable separately, none showed significance. In other words, we observed significance only when both sub-variables were considered as a whole in the same regression block. Once gender roles [*F*_(8, 201)_ = 2.126, *p* = 0.035, *R*^2^ = 0.078] and sexual orientation [F_(9, 200)_ = 2.343, *p* = 0.016, *R*^2^ = 0.095] were included, the final sex*gender model (Model 5) remained significant. A tendency was observed in which non-heterosexual sexual orientations predicted higher performance (*p* = 0.052) in Model 5. Covariates in the final model have increased variance explained [*F*_(16, 193)_ = 1.897, *p* = 0.023, *R*^2^ = 0.130]. Specifically, a younger age significantly predicted better performance (*p* = 0.026). However, adding covariates in the last model did not change the model’s significance.

The model predicting the Verbal Fluency task performances according to birth-assigned sex did not significantly predict performances [*F*_(1, 207)_ = 1.172, *p* = 0.280, *R*^2^ = 0.006]. Adding, respectively, sex hormones [*F*_(4, 204)_ = 0.409, *p* = 0.802, *R*^2^ = 0.008], gender identity [*F*_(6, 202)_ = 0.470, *p* = 0.83, *R*^2^ = 0.014], and gender roles [*F*_(8, 200)_ = 0.455, *p* = 0.887, *R*^2^ = 0.018] in further models also did not significantly predict performances. The final sex*gender model did not explain significant variance, in which none of the incrementally added variables made significant contribution [*F*_(9, 199)_ = 0.410, *p* = 0.929, *R*^2^ = 0.018]. Covariates in the Model 6 did increases variance explained, but this did not attain statistical significance [*F*_(16, 192)_ = 1.100, *p* = 0.358, *R*^2^ = 0.084]. Here, higher drug and alcohol use predicted higher scores (*p* = 0.012).

##### Fine motor skill cognitive task

The model predicting the Purdue Pegboard task performance according to birth-assigned sex explained significant variance. Here, females outperformed males [*F*_(1, 206)_ = 10.007, *p* = 0.002, *R*^2^ = 0.046]. Adding, respectively, sex hormones in Model 2 [*F*_(4, 203)_ = 3.180, *p* = 0.015, *R*^2^ = 0.059] and gender identity in Model 3 [*F*_(6, 201)_ = 2.358, *p* = 0.032, *R*^2^ = 0.066] did not contribute more at explaining performances, but both models remained significant. Once gender roles were included in Model 4 [*F*_(8, 199)_ = 1.868, *p* = 0.067, *R*^2^ = 0.070] and sexual orientation in Model 5 [*F*_(9, 198)_ = 1.652, *p* = 0.103, *R*^2^ = 0.070], both models lost significance and did not significantly predict fine motor skill performances better. Covariates enhanced explained variance and made the final model significant [*F*_(16, 191)_ = 1.889, *p* = 0.023, *R*^2^ = 0.137]. Specifically, younger age significantly predicted better fine motor skill performances (*p* = 0.002).

##### Spatial cognitive tasks

The model predicting the Judgement Line Orientation task performance according to birth-assigned sex explained significant variance. Here, males outperformed females [*F*_(1, 208)_ = 7.508, *p* = 0.007, *R*^2^ = 0.035]. The inclusion of sex hormones in Model 2 increased variance explained and rendered the model even more significant [*F*_(4, 205)_ = 4.098, *p* = 0.003, *R*^2^ = 0.074]. Specifically, lower estradiol (*p* = 0.040) and higher progesterone levels (*p* = 0.032) significantly predicted higher performance. Adding gender identity in Model 3 contributed positively to explain performance and the model remained significant [*F*_(6, 203)_ = 3.927, *p* =  < 0.001, *R*^2^ = 0.104]. Once gender roles were included in Model 4 [*F*_(8, 201)_ = 3.053, *p* = 0.003, *R*^2^ = 0.108] and sexual orientation in Model 5 [F_(9, 200)_ = 2.965, *p* = 0.002, *R*^2^ = 0.118], both models also significantly predicted performances. However, both sub-variables of gender roles showed no significance. In addition, even though model remained significant when sexual orientation was added, only a tendency was observed, where non-heterosexuality could significantly predict better performance (*p* = 0.146). Covariates in the last model increased explained variance, and the model remained significant [*F*_(16, 193)_ = 1.879, *p* = 0.024, *R*^2^ = 0.135]. However, no covariates significantly predicted performance.

The model predicting the Mental Rotation task performance according to birth-assigned sex explained significant variance. Here, males outperformed females [*F*_(1, 208)_ = 9.228, *p* = 0.003, *R*^2^ = 0.042]. Including sex hormones in Model 2 did not explain performance and actually rendered the model non-significant [*F*_(4, 205)_ = 2.370, *p* = 0.054, *R*^2^ = 0.044]. The addition of gender identity in Model 3 [*F*_(6, 203)_ = 1.603, *p* = 0.148, *R*^2^ = 0.045], gender roles in Model 4 [*F*_(8, 201)_ = 1.501, *p* = 0.159, *R*^2^ = 0.056] and sexual orientation in Model 5 [*F*_(9, 200)_ = 1.468, *p* = 0.162, *R*^2^ = 0.062] did not explain performance and all three models remained non-significant. Covariates in the last model increased explained variance but did not make the model significant [*F*_(16, 193)_ = 1.533, *p* = 0.091, *R*^2^ = 0.113].

The model predicting the Rey–Osterrieth Complex Figure task performance according to birth-assigned sex was not significant [*F*_(1, 208)_ = 0.036, *p* = 0.850, *R*^2^ = 0.000]. Adding, respectively, sex hormones [*F*_(4, 205)_ = 0.608, *p* = 0.657, *R*^2^ = 0.012], gender identity [*F*_(6, 203)_ = 0.993, *p* = 0.430, *R*^2^ = 0.029] and gender roles [*F*_(8, 200)_ = 0.808, *p* = 0.596, *R*^2^ = 0.031] in further models also did not significantly add prediction of performance. The final sex*gender model did not explain significant variance, meaning that no sex*gender variable in the hierarchical models significantly predicted performances [*F*_(9, 200)_ = 0.855, *p* = 0.567, *R*^2^ = 0.037]. Covariates in the Model 6 did increase variance explained [*F*_(16, 193)_ = 1.394, *p* = 0.148, *R*^2^ = 0.104]. Specifically, younger age predicted higher scores (*p* = 0.008). Despite this, Model 6 remained non-significant.

##### Immediate digit memory cognitive task

The model predicting the Digit Span task performance according to birth-assigned sex accounted for non-significant variance [*F*_(1, 208)_ = 0.437, *p* = 0.509, *R*^2^ = 0.002]. The further addition of sex hormones [*F*_(4, 205)_ = 0.882, *p* = 0.476, *R*^2^ = 0.017], gender identity [*F*_(6, 203)_ = 1.620, *p* = 0.143, *R*^2^ = 0.046] and gender roles [*F*_(8, 201)_ = 1.408, *p* = 0.195, *R*^2^ = 0.053] in the upcoming models did not significantly predicted performances. The consideration of sexual orientation in the last sex*gender model did not add significant variance [*F*_(9, 200)_ = 1.254, *p* = 0.264, *R*^2^ = 0.053]. Covariates contributed at increasing variance explained in Model 6, but remained non-significant [*F*_(16, 193)_ = 1.219, *p* = 0.256, *R*^2^ = 0.092]. No covariates significantly predicted performances.

##### Figural fluency cognitive task

The model predicting the Five-Point Test performances according to birth-assigned sex was not significant and explained a nearly zero variance explained [*F*_(1, 208)_ = 0.288, *p* = 0.592, *R*^2^ = 0.001]. The incremental inclusion of sex hormones [*F*_(4, 205)_ = 0.742, *p* = 0.564, *R*^2^ = 0.014], gender identity [*F*_(6, 203)_ = 0.663, *p* = 0.680, *R*^2^ = 0.019] and gender roles [*F*_(8, 201)_ = 0.623, *p* = 0.758, *R*^2^ = 0.024] enhanced explained variance, but remained non-significant. The Model 5 did not significantly predict performance [*F*_(9, 200)_ = 0.619, *p* = 0.780, *R*^2^ = 0.027]. The consideration of covariates in Model 6 increased explained variance [*F*_(16, 193)_ = 1.479, *p* = 0.110, *R*^2^ = 0.109]. Specifically, younger age (*p* = 0.007) and a higher drug and alcohol use (*p* = 0.007) significantly explained better performances. However, Model 6 remained non-significant.

## Discussion

This study was interested in deepening the knowledge of SPC over-and-above binary sex by integrating several sex and gender factors together in a sufficiently powered sample. To date, the majority of the SPC studies have considered only birth-assigned sex as their variable of interest. The current study shows the strength of considering all these factors together. In so doing, we aimed to understand the additive effects of sex and gender factors accounted for collectively on SPC. Consistent with existing literature, we partially confirmed our first hypothesis that males would outperform females in spatial tasks, and that females would outperform males in verbal and fine motor skill tasks. We also suggested that SPC would be further influenced by sex hormones. Lastly, we postulated that gender identity, gender roles, and sexual orientation would further explain SPC above and beyond biological factors. Our findings provide partial support for these second hypotheses in that male-typic spatial abilities and fine motor skills were better explained by biological factors while verbal abilities were better explained by socio-cultural gender factors.

### Birth-assigned sex and sexually polymorphic cognition

Our results suggest that there is SPC evidence driven by birth-assigned sex. Indeed, four out of six expected SPC tasks presented sex differences. Birth-assigned males outperformed birth-assigned females on Judgement Line Orientation (η^2^_P_ = 0.032) and Mental Rotation (η^2^_P_ = 0.044), with between small and medium effect sizes. These results are therefore consistent with the ones presented in the meta-analyses by Maeda and Yoon (2013) and Voyer and Voyer (1995) on spatial cognition, which found medium Cohen’s d. Therefore, visuospatial judgement and mental rotation, two components of spatial cognition, exhibit some sexual polymorphism.

On the other hand, birth-assigned females outperformed birth-assigned males on the Perdue Pegboard (η^2^_P_ = 0.049) and on California Verbal Learning Test (η^2^_P_ = 0.020), also with effect sizes ranging between small to medium. Again, both results align with the medium Cohen’s d reported in the literature [35, 183, 184]. Therefore, fine motor skills, verbal memory and verbal learning also exhibit some sexual polymorphism. By contrast, short-term digit memory and figural fluency, both non-expected SPC functions, exhibit no sexual polymorphism.

Interestingly, two expected SPC tasks did not present birth-assigned sex differences. Male–female differences were near-zero in the Rey–Osterrieth Complex Figure test (η^2^_P_ = 0.000) and very small in the Verbal Fluency task (η^2^_P_ = 0.005), as opposed to what is reported in the scientific literature [13, 50, 51]. For starters, the semantic fluency task that was chosen in this study has a female advantage of roughly *d* = 0.11, representing a really small effect [30]. An exploratory power analysis suggested our protocol required a sample size close to *N* = 2000 to find a statistical difference in a one-sided t-test, with two groups, where significance criterion of α = 0.05, and power = 0.80 (as calculated with G*Power version 3.1.9.4). The sample collected seems not to be large enough to get the female advantage statistically significant. However, descriptively, the advantage (η^2^_P_ = 0.005) is present and might very well be around *d* = 0.11, given that both effects are interpreted as really small. On the other hand, the effect of sex in Rey–Osterrieth Complex Figure performances seems to be non-existent in this sample. Although the Rey–Osterrieth Complex Figure Test measures spatial memory, it is at its core a drawing task and therefore an arguably female-typic gendered creative activity [3]. This type of activity might correlate with feminine gender roles and will benefit from further study of the influence of different sex and gender factors on its performance. Despite Rey–Osterrieth Complex Figure, our results show the relevance of considering spatial and verbal cognition as having several distinct cognitive functions.

Measuring SPC exclusively through birth-assigned sex is the general tendency in the SPC literature. However, our results provide clear evidence that sexual differences according to birth-assigned sex exists, albeit of relatively small to medium effect sizes. Notwithstanding, effect sizes observed for male-typed tasks were higher than those observed for female-typed tasks. This could be explained by the fact that most of the gender-diverse participants were assigned female at birth (49 females and 17 males). The consideration of gender identity in SPC study has provided more solid foundation on birth-assigned sex’s impact on SPC. Even though one-third of our sample (*n* = 66) is gender diverse, some male–female differences in spatial cognition, verbal cognition, and fine motor skills remain. Despite this, human experience goes far beyond birth-assigned sex, and considering this variable as able to capture all sex*gender factors and their interconnected complexity is a non-negligible limitation. This holistic and transdisciplinary research approach is the key strength of our methodology.

### Sociocultural gender and sexual orientation on sexually polymorphic cognition

The SPC literature has shown the importance of socio-cultural gender factors like gender identity, gender roles [8, 100], and sexual orientation [125]. To reiterate, sexual orientation is orthogonal to gender identity and gender roles [116, 117]. Therefore, we use the term *socio-cultural gender and sexual orientation* (SGSO) in our discussion to demarcate this distinction. We expected that SGSO variables would further explain SPC, especially given the sexual and gender diversity of our sample. Indeed, 63% of our analytic sample identified as members of the LGBTQ+ community. Consistent with our hypothesis, these socio-cultural factors improved the percentage of variance explained by our main regression models that also included birth-assigned sex and sex hormones. This means that SGSO factors contributed additively in their own unique variance explained in SPC. When considered collectively, gender identity, gender roles, and sexual orientation as explanatory variables of SPC exhibited variations based on the specific cognitive functions involved.

Verbal memory appears to be more influenced by SGSO factors than by biological sex-based factors. In fact, the CVLT results showed a percentage of variance explained by these factors of 5.0%, a higher percentage than that observed for the spatial cognitive tasks. Perhaps most interestingly, the 5.0% of variance explained by these SGSO factors is slightly greater than the 4.5% explained by the biological factors. Although verbal fluency shows a higher impact of these three psychosocial factors (1.0% of variance explained) than biological factors (0.8%), the sexual polymorphism of this cognitive function is rather negligible (no significant differences were observed in each model). Even though this result appears to align with those of verbal memory, the lack of significance makes it difficult to include it into this interpretation. It also merits mention that obtained effect sizes are quite low. With this in mind, for verbal cognitive functions which present a female advantage according to birth-assigned sex, sexual polymorphism is mainly driven by the "psychosocial gender" aspect, making this cognitive sphere potentially more gendered than sexed.

By contrast, some spatial abilities appear to be less influenced by socio-cultural gender than by biological sex-based factors. Visuospatial judgment and mental rotation had a cumulative percentage of variance explained by these three factors of, respectively, 4.4% and 1.8%. When compared with the percentages of variances explained by biological factors (birth-assigned sex and sex hormones), which were, respectively, 7.4% for visuospatial judgment and 4.4% for mental rotation, it is possible to highlight the greater impact of biological factors compared with socio-cultural factors for these cognitive abilities. Therefore, for spatial cognitive functions with a male advantage based on birth-assigned sex, sexual polymorphism seems to be supported mainly by the "biological" side, i.e., the portion that is more sexed than gendered.

**Fig. 4:**
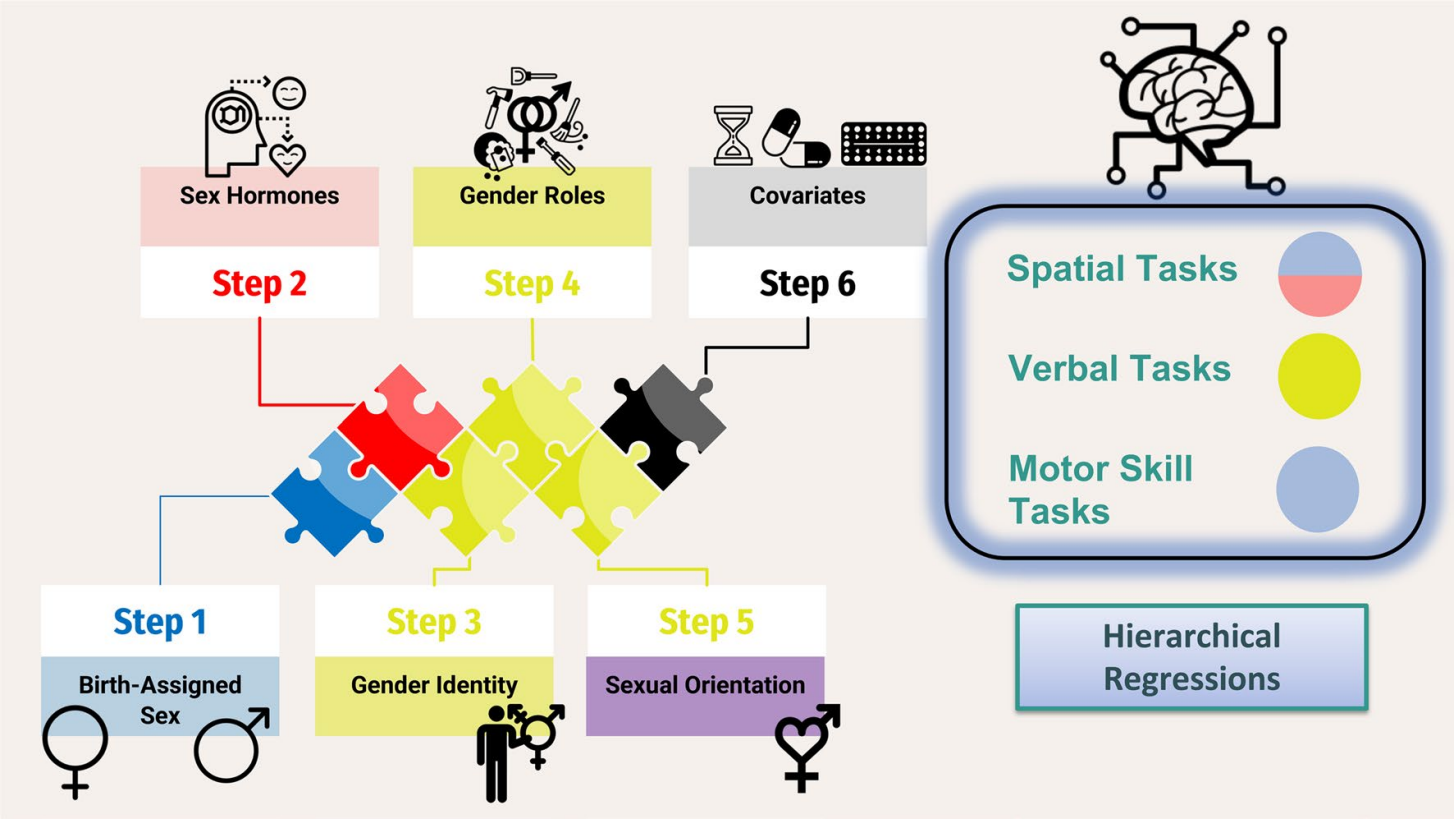
Overall contribution of biological and psychosocial sex and gender factors in the different cognitive spheres

Fine motor skills showed preliminary male–female differences. Unlike verbal cognition, SGSO factors contributed to explain only 1.1% the motor side of SPC. Biological factors explained 5.9%, i.e., more than five times the variance explained by SGSO factors. Moreover, birth-assigned sex alone explained 4.6%, showing a greater importance of this variable than SGSO factors for motor cognition. The sexual polymorphism of motor cognition seems to be induced more by its biological side, making this cognitive function more sexed than gendered. This result could be explained by looking at the sensitive period of development for fine motor skills that occurs during childhood. Indeed, a study published by D. Watanabe and colleagues suggest the presence of a developmental window that coincide with the start of school age [185]. After this sensitive period, fine motor skills could be less impacted by environmental factors. Our hypothesis that SGSO factors contribute to better explain SPC than sex alone is therefore partially confirmed. A visual illustration of sex and gender factors’ contribution is represented by Fig. 4.

Although sex and gender factors other than birth-assigned sex did not strongly predict fine motor performance, age proved to be a highly significant predictor, in line with what has been observed in the literature [186, 187]. In the same way as birth-assigned sex and sex hormones, age can be considered a biological variable [188]. Sex hormones interact with age and should be considered in future SPC studies [10]. Taken together, these results indicate and suggest that SPC manifests itself from different combinations of factors.

The consideration of chronological age brings temporality and is inherent to the whole developmental paradigm in cognitive research. This perspective provides a common thread that can explain the complex interweaving of sex and gender factors. Our results suggested that spatial cognition and fine motor skills are cognitive functions for which their sexual polymorphism is biologically driven. These results correlate well with the earlier onset of their sexual differences. Literature shows that girls outperform boys in fine motor skills from the age of five when they start school [31, 189]. Similarly, studies have shown that young boys start performing better in spatial cognition from school age onward [190], and even before [191, 192]. The earlier differentiation of these cognitive functions could explain the more “prominent” contribution of biological factors, which appear earlier on in development [193]. Future research should explore potential sex by age interaction effects to understand this better.

On the other hand, sex differences in verbal memory tend to be small in childhood and in old age, but higher in early adulthood [13]. The performance gap favoring women widens from adolescence onwards. The later differentiation of these cognitive functions could be explained by the contribution of these SGSO factors, which appear in early adolescence but continue to develop and consolidate into adulthood. Accordingly, these results highlight the importance of considering sex as a biological factor and gender as a socio-cultural factor both, respectively, influencing cognitive functioning across lifespan development.

### Sex hormones and sexually polymorphic cognition

Based on our previous interpretations and expectations [19, 131, 194], a major hypothesis of this study was that sex hormones would have a greater impact on SPC than birth-assigned sex. Similar in spirit to the unique influence of SGSO factors, this hypothesis is only true for certain cognitive abilities in our study. Indeed, the various hierarchical multiple regression models highlighted different pathways for the involvement of different sex and gender factors according to cognitive function. For example, spatial cognition seemed to be more affected by biological factors than verbal and motor cognition. However, when we look at the improvement in model fit with the addition of sex hormones, the impact of sex hormones was notable not only for the visuospatial judgement task, but also for verbal memory. The verbal memory task showed significance for lower estradiol levels (*p* = 0.041) and a significant trend for higher testosterone levels (*p* = 0.080). The visuospatial judgment task showed significant effects for lower estradiol levels (*p* = 0.040) and for higher progesterone levels (*p* = 0.032). Mental rotation performance was predominantly mediated by sex assigned at birth but was influenced very little by sex hormones.

In our regression models, the first block was composed of birth-assigned sex while the second block added sex hormones. Consequently, the impact of the variables included in the second block was controlled by the variance in the first block. In other words, males have higher testosterone levels than females, and block 2 adjusts for the variance explained by this result. The fact that estradiol levels were a significant predictor of the visuospatial judgment task is therefore not out of the ordinary [52]. More interestingly, low estradiol and high testosterone levels seemed to predict verbal memory performance, when controlled for birth-assigned sex. This suggests that the effect of sex hormones seems to be sex-dependant, but in the opposite direction to that expected, when variance explained by birth-assigned sex is adjusted for. Based on this, we could hypothesize an inverted U-shaped curve, suggesting better performances in verbal memory when there is an optimal level of both estradiol and testosterone. Similarly, higher levels of progesterone seem to predict male-typed visuospatial judgement.

Although the scientific literature suggests that high levels of testosterone are linked to better performance on spatial tasks and high levels of estradiol are linked to verbal tasks, there are some studies that have highlighted the presence of an inverted U-shaped curve for testosterone and spatial tasks [52, 195]. A study published in 2021 by Sankar and Hampson even suggested a similar inverted U-shaped curve for estradiol in SPC. Our results reinforce this hypothesis. Higher estradiol in a male-typed task and lower estradiol, as well as higher testosterone in a female-typed task, predicted better respective performance. This suggests that optimal and balanced levels of testosterone and estradiol both in male-typed and female-typed task could better explain cognitive performance beyond having, for example, high estradiol levels alone in verbal tasks or high testosterone levels alone in spatial tasks. Being antagonistic hormones, this equilibrium would be represented by the apex of the U-shaped curve [196]. To summarize, cognitive performance in verbal memory and visuospatial judgment seems to be impacted by sex hormones in an inverted U-shaped way. However, the absence of significant sex hormone impacts for the other SPC tasks could be because the variance of sex hormones is very strongly controlled by the variance of sex assigned at birth.

### Final models and covariates of sexually polymorphic cognition

The final models comprised nine factors, making up the first five blocks, plus a final block that included seven covariates. This final addition of covariates in a last block rather than as a first block was intended to clearly observe the impacts of the different sex and gender factors of interest unadjusted for covariates. Moreover, if the variance explained by the covariates was considerable when compared with that of the various sex and gender factors, we could interpret better whether these cognitive functions were sexually polymorphic or not. This being said, there are potential interaction effects (e.g., sex X age) that could be explored in the future with more power and where we would want to adjust for covariates in the first models as is more traditionally the case.

SPC tasks were predicted more by sex and gender factors than by covariates. Verbal memory performance was at 9.5% predicted by sex and gender factors, compared to 3.5% by covariates, almost 3X higher. Similar ratios were observed for the three other sexually polymorphic tasks (see Tables 4, 5, 6, 7, 8, 9). For the four remaining tasks that were not sexually polymorphic, the importance of covariates was greater than sex and gender factors’, as observed in verbal fluency, where sex and gender factors predicted 1.8%, compared to 6.6% by covariates (see Tables 4,5, 6, 7, 8, 9). This underlines that non-SPC tasks differ according to several variables, and therefore display a certain variability among individuals.

As expected from the scientific literature [197–199], older age proved to be a significant covariate in explaining decrements in verbal memory, spatial memory, motor skills, and figural fluency. In addition, a higher frequency of substance use behaviors, a covariate that was added to the model a posteriori informed by preliminary analyses (see section Statistical analyses), had a significant impact on cognitive performance in verbal and figural fluency (see Tables 4, 5, 6, 7, 8, 9). The fluency aspect of cognition may stimulate via neural mechanisms underlying that stimulated by alcohol, cannabis, and illicit drug use. Given that no study has examined the subject, this result could provide a rationale for investigating this link between alcohol and drug use and verbal and figural fluency. Taken together, consideration of certain covariates enabled us to ensure a certain variability in the performances observed, while allowing us to examine the direct effect of sex and gender factors of interest in cognition.

In sum, SPC appears to be impacted by sex and gender factors via different pathways depending on the specific cognitive function examined. Although the results obtained considered both sex as a biological factor and gender as a socio-cultural factor, it is crucial to acknowledge the bi-directional association of these sex and gender factors. Studies showing that gender-based factors impact cognition by an inter-correlation with biological factors [22, 200]. Certain critical periods in the development of biological factors of SPC occur at the same time as the emergence of some gender-based factors [201]. The period around the sixth month of life is characterized by a high exposure of sex hormones, resulting in permanent and irreversible effects [202]. During this period, there is a preference for objects that correspond to our gender [203]. Similarly, the pubertal period when sex hormones have a neuropsychological activation role rather than neuroanatomical one corresponds to the time when sexual orientation is at its peak of questioning [204, 205]. Therefore, there is a possibility that bi-directional links between sex and gender factors could lead to a better understanding of SPC. However, with a cross-sectional design (measures collected at one time-point), it is impossible to infer the direction of the relationship. More studies are needed to understand the temporal influence of these factors.

### Strengths and limitations

The integration and consideration of both sex and gender factors both collectively and, respectively, makes this a pioneering study in the world of SPC. The quasi-experimental protocol overcame two limitations of previous studies: namely, the consideration of sex from a purely binary point of view and the consideration of only one sex and gender factor at a time, thus limiting understanding of the relationship between these different factors. To achieve this, we were able to recruit 74 sexually diverse people and 66 gender-diverse people. Not only is this one of the few studies to have been able to recruit so many people from this community, but this large proportion of LGBTQ+ people allowed us to have a high representativeness of the sex and gender factors of interest. This therefore enabled us, from a statistical point of view, to measure the importance of gender identity and sexual orientation.

The protocol also included eight cognitive tasks. These tasks focused on SPC functions and aimed to consider a range of cognitive abilities, including verbal and spatial memory, memory span, mental rotation, visuospatial judgment, fine motor skills and peripheral assessment of certain executive functions. Therefore, it is highly probable that participants, having reached a certain point in the protocol, are subject to cognitive fatigue. Bearing in mind that each participant has his or her own fatigue threshold, and that its attainment varies greatly depending on the time between 12AM and 5PM at which the tasks were performed, this constitutes a limit to the generalizability of the results. Moreover, rather than a fatigue effect, participants could have been subject to cognitive habituation, given the number of tasks they were asked to complete. However, the temporal arrangement of the different cognitive tasks was carefully considered and organized in such a way as to minimize overlap between the cognitive functions involved, thus limiting the possibilities of habituation or localized cognitive fatigue. In addition, for ethical purposes, participants were informed that SPC was the subject of the study and might expect to be presented cognitive tasks that are performed differently between men, women, and gender-diverse people. To overcome this possible bias, the protocol included two tasks measuring non-SPC functions. Having observed no differences on these tasks, the ones observed in SPC tasks were unlikely to be driven by the foreknown research subject.

This study nevertheless has several limitations. Because we had eight cognitive tasks to observe, we had 8 hierarchical regression models, each comprising a total of 16 variables. Although our sample size was not a problem according to the power analysis performed, it did not consider the number of dependent variables and multiple comparisons we had. The same participants, with the same demographic characteristics and the same sex and gender profiles, were considered in these analyses eight times. Consequently, a concern for generalizability must be taken into consideration. Moreover, the protocol for this study lasted around 130 min (*M* = 128.18, SD = 21.30). Consequently, the performance of eight cognitive tasks, although organized in such a way as to limit fatigue, most certainly contribute to cognitive fatigue for some participants. An experimental protocol of shorter duration, or split into two sessions close in time, could limit this fatigue effect.

Some cognitive tasks were designed for neuropsychological diagnostic purposes. This includes the Judgement Line Orientation and the Rey–Osterrieth Complex Figure. More specifically, these tasks were generally administered to children during neurodevelopmental assessments, or to adults during assessments of cognitive decline. In this sample, the average age was in the "young adult” range and only few of them had neuropsychological diagnosis. As the means were quite high, the low variability in the scores resulting from the negative asymmetry in performance may limit the generalizability of the results to the general population. Therefore, although our postulates of normality and homoscedasticity were respected, it is important to consider this highly skewed distribution of cognitive performance. With regard to the verbal fluency task used, it seemed to be more akin to categorical or semantic fluency, as previously stated by the meta-analysis conducted by Hirnstein et al. [30]. Indeed, these authors suggest that women's better performance would be mainly observed in phonemic fluency and certain categories of semantic fluency. Perhaps SPC results in verbal fluency could have been investigated more easily if the task chosen was more akin to phonemic fluency.

Furthermore, the psychosocial measures chosen to assess SPC have certain limitations. Indeed, the variable used to measure gender roles was the controversial Bem questionnaire assessing the levels of masculinity and femininity. Given that gender roles go far beyond gendered personality traits, we cannot claim that this is a holistic and comprehensive measure of gender roles. We recommend that future studies take into consideration other facets of gender roles, such as the distribution of household tasks, career choices, level of involvement in child-rearing, and other gendered behaviors. In addition, the measure of sexual orientation used was a dichotomous application of the classic Kinsey scale. This scale was designed to measure sexual orientation from a dimensional perspective. However, we put this scale back into a categorical form to comprise the "non-heterosexual" category. Given that we wanted to include asexual people in the analyses and that the scale did not present a measure for asexuality, we decided to include them in this measure of "non-heterosexuality". Sexual orientation was therefore not considered in a dimensional approach as originally intended, thus losing a certain richness of data.

Finally and most importantly, although our study sought to adopt a transdisciplinary perspective to overcome the "silo effect" that has commonly impacted studies in the field of SPC, it does not measure the interaction effects that exist between the various factors. In fact, the statistical power we had did not allow us to investigate certain interaction phenomena considered primordial in cognitive research, such as between sex and age, or between sex hormones and sex assigned at birth. We acknowledge this limitation and suggest research that has more power to investigate these interactions. Over and above statistical interactions, developmental interaction could not be investigated. Indeed, the critical developmental period of sex hormones coincide with the time when gender roles begin to impact gendered conceptions. Similarly, the critical developmental period of gender identity, around puberty, also corresponds to the time when sexual orientation develops, and the body undergoes major hormonal changes [19]. That said, while it is interesting to consider several factors in a single study, this methodology fails to capture the developmental interactions that exist between variables on the neuroendocrine and psychosocial sides. A new transdisciplinary approach complementary to the one used here would therefore be very useful to deepen our knowledge of sex and gender correlates vis-à-vis sex differences in cognitive functioning.

### Perspective and significance

In summary, our findings suggest that SPC is influenced by a multitude of interacting sex and gender factors that modulate pathways in diverse ways. Four of the six SPC tasks in our protocol were in fact sexually polymorphic. Verbal fluency and spatial memory were not sexually polymorphic. Our comprehensive consideration of different socio-cultural gender factors and sexual orientation also enabled better prediction of cognitive performance in SPC tasks, especially for verbal memory. Spatial cognition, on the other hand, was predicted more by biological sex-based factors, as were fine motor skills. The importance of sex hormones, although overlapping with by birth-assigned sex, helped generate thinking regarding an inversed U-shaped curve for cognitive performance and sex hormones.

## Conclusion

This study helps advance our understanding of SPC by the consideration of both biological and psychosocial perspectives. Our holistic and integrative research approach provides strong support for the integration of both sex and gender measures in further SPC research. Permit us to highlight two key takeaways. First, certain tasks were better predicted by gender identity than by birth-assigned sex. This underlines the importance of considering gender diversity when seeking to understand sex differences and gender diversity in cognition. Second, our multidimensional statistical approach complements unidimensional studies that consider sex and gender variables separately. Notwithstanding, our cross-sectional design cannot infer the developmental dynamics that may influence of these different factors. Developmental studies are therefore needed in future enrich SPC research.

### Supplementary Information

**Additional file 1. Table S1:** Correlation matrix of cognitive task performances for participants. **Table S2:** Regression table of Digit Span performances according to all five sex*gender factors and covariates. **Table S3:** Regression table of Five-Point Test performances according to all five sex*gender factors and covariates.

## Data Availability

All data have been collected via participants recruited in this study. Databases will be available upon request.
