# Supplementary material for: Sex and gender correlates of sexually polymorphic cognition
Source: Biol Sex Differ. 2024 Jan 8;15:3. doi: 10.1186/s13293-023-00579-8 (PMC10773055; doi:10.1186/s13293-023-00579-8)
Supplement: Supplementary file 1 — Additional file 1. Table S1: Correlation matrix of cognitive task performances for participants. Table S2: Regression table of Digit Span performances according to all five sex*gender factors and covariates. Table S3: Regression table of Five-Point Test performances according to all five sex*gender factors and covariates. [file 13293_2023_579_MOESM1_ESM.docx]

**Additional file**

**Table S1: Correlation matrix of cognitive task performances for participants**

| Variables | 1 | 2 | 3 | 4 | 5 | 6 | 7 | 8 | 9 |
| --- | --- | --- | --- | --- | --- | --- | --- | --- | --- |
| 1. Digit Span | — |  |  |  |  |  |  |  |  |
| 1. California Verbal Learning Test | 0.224^***^ | — |  |  |  |  |  |  |  |
| 1. Judgement Line Orientation | 0.194^**^ | 0.328^***^ | — |  |  |  |  |  |  |
| 1. Mental Rotation | 0.032 | 0.132^*^ | 0.200^**^ | — |  |  |  |  |  |
| 1. Rey–Osterrieth Complex Figure (Immediate Recall) | 0.077 | 0.309^***^ | 0.299^***^ | 0.333^***^ | — |  |  |  |  |
| 1. Rey–Osterrieth Complex Figure (Delayed Recall) | 0.063 | 0.298^***^ | 0.302^***^ | 0.291^***^ | 0.960^***^ | — |  |  |  |
| 1. Purdue Pegboard Test (Sum of Three First Trials) | 0.063 | 0.260^***^ | 0.072 | 0.033 | 0.090 | 0.081 | — |  |  |
| 1. Purdue Pegboard (Assembly Trial) | 0.065 | 0.351^***^ | 0.196^**^ | 0.174^**^ | 0.182^**^ | 0.183^**^ | 0.577^***^ | — |  |
| 1. Verbal Fluency (Total Score) | 0.291^***^ | 0.467^***^ | 0.317^***^ | 0.184^**^ | 0.261^***^ | 0.246^***^ | 0.163^*^ | 0.271^***^ | — |
| 1. Five-Point Test | 0.103 | 0.224^***^ | 0.276^***^ | 0.199^**^ | 0.337^***^ | 0.346^***^ | 0.222^***^ | 0.377^***^ | 0.227^***^ |

Note: * p < 0.05; ** p < 0.01; *** p < 0.001.

**Table S2. Regression table of Digit Span performances according to all five sex*gender factors and covariates**

| **Predictor Factors** | | R^2^ | R^2^ Adjusted | ΔR^2^ | p | B | SE B | β | t | p | 95% CI: LL | 95% CI: UL |
| --- | --- | --- | --- | --- | --- | --- | --- | --- | --- | --- | --- | --- |
| **Model 1** |  | 0.002 | -0.003 | 0.002 | 0.509 |  |  |  |  |  |  |  |
| Birth-Assigned Sex (Male = 0, Female = 1) | | |  |  |  | -0.098 | 0.148 | -0.046 | -0.661 | 0.509 | -0.389 | 0.193 |
| **Model 2** |  | 0.017 | -0.002 | 0.015 | 0.476 |  |  |  |  |  |  |  |
| Testosterone | |  |  |  |  | -0.002 | 0.001 | -0.124 | -1.526 | 0.128 | -0.004 | 0.001 |
| Estradiol |  |  |  |  |  | -0.081 | 0.170 | -0.037 | -0.479 | 0.632 | -0.417 | 0.254 |
| Progesterone | |  |  |  |  | 0.000 | 0.001 | -0.008 | -0.104 | 0.917 | -0.002 | 0.002 |
| **Model 3** |  | 0.046 | 0.018 | 0.029 | 0.143 |  |  |  |  |  |  |  |
| Gender Identity, Woman (Not a Woma*n =* 0, Woman = 1) | | | |  |  | -0.145 | 0.288 | -0.065 | -0.504 | 0.614 | -0.714 | 0.423 |
| Gender Identity, GD (Not GD = 0, GD = 1) | | | |  |  | 0.328 | 0.247 | 0.135 | 1.329 | 0.185 | -0.158 | 0.814 |
| **Model 4** |  | 0.053 | 0.015 | 0.007 | 0.195 |  |  |  |  |  |  |  |
| BEM Femininity | |  |  |  |  | -0.070 | 0.109 | -0.046 | -0.641 | 0.523 | -0.286 | 0.146 |
| BEM Masculinity | |  |  |  |  | 0.111 | 0.098 | 0.081 | 1.140 | 0.255 | -0.081 | 0.304 |
| **Model 5** |  | 0.053 | 0.011 | 0.000 | 0.264 |  |  |  |  |  |  |  |
| Sexual Orientation (0 = Heterosexual, 1 = Non-heterosexual) | | | | |  | 0.047 | 0.169 | 0.022 | 0.280 | 0.780 | -0.286 | 0.381 |
| **Model 6 : Covariates** | | 0.092 | 0.016 | 0.038 | 0.256 |  |  |  |  |  |  |  |
| Age |  |  |  |  |  | -0.002 | 0.010 | -0.014 | -0.189 | 0.851 | -0.021 | 0.017 |
| DHEA/Cortisol Ratio | |  |  |  |  | <0.001 | 0.000 | -0.015 | -0.202 | 0.840 | 0.000 | 0.000 |
| Language (0 = French, 1 = English) | |  |  |  |  | 0.310 | 0.224 | 0.104 | 1.384 | 0.168 | -0.132 | 0.752 |
| Hormone and Surgery Index | |  |  |  |  | -0.150 | 0.188 | -0.065 | -0.798 | 0.426 | -0.521 | 0.221 |
| Contraceptive Use (0 = No Contraceptive, 1 = Contraceptive) | |  |  |  |  | -0.391 | 0.217 | -0.146 | -1.803 | 0.073 | -0.819 | 0.037 |
| Substance Use Index | |  |  |  |  | 0.031 | 0.059 | 0.038 | 0.529 | 0.598 | -0.085 | 0.148 |
| Physical and Mental Health Index | | |  |  |  | -0.126 | 0.103 | -0.091 | -1.229 | 0.221 | -0.330 | 0.007 |

**Table S3. Regression table of Five-Point Test performances according to all five sex*gender factors and covariates**

| **Predictor Factors** | | R^2^ | R^2^ Adjusted | ΔR^2^ | p | B | SE B | β | t | p | 95% CI : LL | 95% CI : UL |
| --- | --- | --- | --- | --- | --- | --- | --- | --- | --- | --- | --- | --- |
| **Model 1** |  | 0.001 | -0.003 | 0.001 | 0.592 |  |  |  |  |  |  |  |
| Birth-Assigned Sex (Male = 0, Female = 1) | | | |  |  | -0.373 | 0.695 | -0.037 | -0.537 | 0.592 | -1.743 | 0.997 |
| **Model 2** |  | 0.014 | -0.005 | 0.013 | 0.564 |  |  |  |  |  |  |  |
| Testosterone | |  |  |  |  | 0.000 | 0.006 | 0.003 | 0.036 | 0.971 | -0.011 | 0.011 |
| Estradiol |  |  |  |  |  | -0.857 | 0.801 | -0.083 | -1.070 | 0.286 | -2.436 | 0.722 |
| Progesterone | |  |  |  |  | 0.007 | 0.005 | 0.123 | 1.545 | 0.124 | -0.002 | 0.017 |
| **Model 3** |  | 0.019 | -0.010 | 0.005 | 0.680 |  |  |  |  |  |  |  |
| Gender Identity, Woman (Not a Woman: 0, Woman: 1) | | | |  |  | -1.387 | 1.375 | -0.131 | -1.009 | 0.314 | -4.097 | 1.324 |
| Gender Identity, GD (Not GD = 0, GD = 1) | | | |  |  | -0.793 | 1.176 | -0.069 | -0.675 | 0.501 | -3.112 | 1.525 |
| **Model 4** |  | 0.024 | -0.015 | 0.005 | 0.758 |  |  |  |  |  |  |  |
| BEM Femininity | |  |  |  |  | -0.395 | 0.523 | -0.055 | -0.755 | 0.451 | -1.426 | 0.636 |
| BEM Masculinity | |  |  |  |  | 0.354 | 0.466 | 0.055 | 0.759 | 0.448 | -0.565 | 1.273 |
| **Model 5** |  | 0.027 | -0.017 | 0.003 | 0.780 |  |  |  |  |  |  |  |
| Sexual Orientation (0 = Heterosexual, 1 = Non-heterosexual) | | | | |  | -0.624 | 0.806 | -0.061 | -0.744 | 0.440 | -2.214 | 0.966 |
| **Model 6: Covariates** | | 0.109 | 0.035 | 0.082 | 0.110 |  |  |  |  |  |  |  |
| Age |  |  |  |  |  | -0.120 | 0.044 | -0.206 | -2.708 | 0.007 | -0.208 | -0.033 |
| DHEA/Cortisol Ratio | |  |  |  |  | -0.001 | 0.000 | -0.099 | -1.377 | 0.170 | -0.001 | 0.000 |
| Language (0 = French, 1 = English) | | |  |  |  | -0.841 | 1.044 | -0.060 | -0.806 | 0.421 | -2.899 | 1.217 |
| Hormone and Surgery Index | |  |  |  |  | 0.118 | 0.876 | 0.011 | 0.134 | 0.893 | -1.609 | 1.844 |
| Contraceptive Use (0 = No Contraceptive, 1 = Contraceptive) | | | | |  | 0.698 | 1.011 | 0.055 | 0.690 | 0.491 | -1.296 | 2.692 |
| Substance Use Index | |  |  |  |  | 0.751 | 0.275 | 0.196 | 2.732 | 0.007 | 0.209 | 1.293 |
| Physical and Mental Health Index | | |  |  |  | 0.253 | 0.480 | 0.039 | 0.528 | 0.598 | -0.693 | 1.199 |
